# Environmental responsiveness of flowering time in cassava genotypes and associated transcriptome changes

**DOI:** 10.1371/journal.pone.0253555

**Published:** 2021-07-21

**Authors:** Deborah N. Oluwasanya, Andreas Gisel, Livia Stavolone, Tim L. Setter

**Affiliations:** 1 Section of Soil and Crop Sciences, School of Integrative Plant Science, Cornell University, Ithaca, New York, United States of America; 2 Bioscience Unit, International Institute of Tropical Agriculture, Ibadan, Oyo State, Nigeria; 3 Institute for Biomedical Technologies, National Research Council (CNR), Bari, Italy; 4 Institute for Sustainable Plant Protection, National Research Council (CNR), Bari, Italy; Shanghai Institutes for Biological Sciences, CHINA

## Abstract

Cassava is an important food security crop in tropical regions of the world. Cassava improvement by breeding is limited by its delayed and poor production of flowers, such that cassava flowering under field conditions indirectly lengthens the breeding cycle. By studying genotype and environment interaction under two Nigerian field conditions (Ubiaja and Ibadan) and three controlled temperature conditions (22°C/18°C, 28/24°C and 34/30°C (day/night)), we found that while early flowering genotypes flowered at similar times and rates under all growing conditions (unfavorable and favorable field and controlled-temperature environments), late flowering genotypes were environmentally sensitive such that they were substantially delayed in unfavorable environments. On the basis of nodes-to-flower, flowering of late genotypes approached the flowering time of early flowering genotypes under relatively cool Ubiaja field conditions and in growth chambers at 22°C, whereas warmer temperatures elicited a delaying effect. Analysis of transcriptomes from leaves of field and controlled-temperature environments revealed that conditions which promote early flowering in cassava have low expression of the flowering repressor gene *TEMPRANILLO 1* (*TEM1*), before and after flowering. Expression data of field plants showed that the balance between flower stimulatory and inhibitory signaling appeared to correlate with flowering time across the environments and genotypes.

## Introduction

Cassava (*Manihot esculenta* Crantz) is a tropical plant originating from the Amazonian region of South America, which is currently cultivated in tropical regions throughout the world for its starchy storage roots [1]. It is an important staple food in the tropics and ranks as the fifth most important source of starch in the world [2]. Cassava improvement has recently received renewed attention with major projects to investigate its source-sink relationships [3], its photosynthetic efficiency [4], the potential use of genomic selection in breeding [5], and expanded scope of target traits for breeding [6], including end-product quality traits [7]. Although it can be propagated asexually, to develop improved cultivars through breeding requires sexual reproduction and associated genetic recombination and selection for genetically superior traits [8].

Sexual reproduction in cassava is limited at multiple phenological stages ranging from the transition to flowering to the development of fruits and seeds [9–13]. Flowering time is a critical factor in cassava breeding because it determines, in part, the length of the breeding cycle, and hence, the rate of genetic improvement. Cassava breeding programs usually begin crossing at 2.5 months after planting (MAP), and most crossing is done at around 4 to 5 MAP, but some crossing continues much later. The development of new cultivars takes eight to ten years due to several obstacles including the difficulty of obtaining sufficient progeny for phenotyping, and late flowering of many cassava lines [8,14]. Each breeding cycle of crossing and selection can be delayed for an additional year or more in desirable, late flowering germplasm, caused by delayed flowering [8]. Some highly desirable genotypes do not flower at all in environments where crossing is to be performed [8,15]. Furthermore, breeding programs that use genomic selection, whereby individuals can be selected in early stages after a cross without field phenotypic evaluation, would benefit if crosses were possible in younger plants, such that cassava selection cycles could be reduced from 4–5 years to 2 years [5,14].

Floral initiation in cassava is associated with fork-type branching [10]. Two types of branching occur in cassava: lateral branching involving buds in the axils of leaves, and apical fork-type branching. Inflorescences always develop at the apex of the developing stem by floral initiation and conversion of the apical meristem to an inflorescence meristem. Following floral initiation at the shoot apex, two to four buds beneath the inflorescence are released from inhibition and undergo sympodial stem development to form branches and a fork. Every flowering event, therefore, results in branching. These fork-type branches at tier 1 each bear new shoot apical meristems, which develop stems with several internodes/nodes containing leaves, until the process of floral initiation repeats at their apices, which are called tier 2, and the process repeats to form subsequent tiers.

Floral induction is regulated by environmental cues (such as temperature and photoperiod) which ensure that flowering occurs under the most optimal conditions for reproductive success [16]. The role of temperature in regulating flowering time is particularly important for cassava that is grown in the tropics where daylengths do not vary significantly throughout the year. In cassava, flowering time is favored by long days and relatively cool (but not vernalization) temperatures [11]. This is in contrast to the model plant *Arabidopsis thaliana*, in which the time to flowering is hastened in warmer ambient temperatures [17], although it also flowers in response to long days (short nights) [18]. The genetic control of flowering time has been well characterized in *A*. *thaliana* and over 300 genes have been identified by forward and reverse genetics to be involved in flowering time regulation, as documented in the flowering database FLOR-ID [19]. The *FLOWERING LOCUS T* (*FT*) gene has been shown to be a flowering integrator of multiple flower inductive pathways and is positively correlated with flowering time in most species studied so far [20]. In cassava, as in many other species, the overexpression of the Arabidopsis *FT* gene and native cassava *FT* gene has been shown to accelerate flowering time in otherwise very late flowering genotypes [9,21,22]. This provides strong evidence for the involvement of *FT* homologs in regulating flowering time in cassava. In Arabidopsis, warm temperatures are favorable for flower induction and, correspondingly, *FT* expression is elicited by long days and warmer temperatures [17]. In cassava, which has two homologs of *FT*, *MeFT1* and *MeFT2* [11], flowering is stimulated by long days, and correspondingly, long days elicit expression of *MeFT2* at the end of long days. However, while cool temperatures are favorable to flower induction, increased expression of *MeFT1* and *MeFT2* in response to cool temperature is not consistent among genotypes, suggesting that other signaling factors, such as inhibitory factors, might be involved [11].

Researchers at the International Institute of Tropical Agriculture (IITA), Ibadan, Nigeria had previously identified a field location (Ubiaja, Nigeria) where flowering occurs earlier and general flower development is enhanced [23,24], independently of the soil characteristics of the two environments [24]. A limited set of weather data suggested that temperature is generally cooler in Ubiaja than Ibadan [25], consistent with cassava’s earlier flowering in Ubiaja.

To advance our understanding of factors influencing cassava flowering, we compared the flowering behaviors of eight genotypes (representing a range of flowering times) under the ‘favorable’ Ubiaja field and the ‘unfavorable’ Ibadan field conditions. In parallel, under controlled conditions, we studied the effect of temperatures ranging from 22°C to 34°C on the flowering times of three of these genotypes. We analyzed the transcriptome of a sub-set of genotypes in these field environments, and in growth chamber conditions at three controlled temperatures. Our findings indicated that the expression of a group of flowering-related genes is consistently regulated under favorable and unfavorable flowering conditions in the field and at the tested temperatures.

In the face of challenges due to global climate change, advancing our understanding of the molecular basis of flowering-time control in cassava is valuable to enhance cassava breeding for crop improvement and opens new possibilities to develop strategies and methodologies to allow cassava flowering irrespective of the environmental growth conditions.

## Materials and methods

### Plant materials and growing conditions

#### (a) Field station

Field experiments were conducted from June 2017 to January 2018 at two field stations in Nigeria: Ibadan (7.4° N and 3.9°E, 230 m asl) in Oyo State and Ubiaja (6.6° N and 6.4° E, 221 m asl) in Edo State. At each location the land was tilled and ridged with no extra nutrients or soil amendments added. Fields were kept free of weeds with hand weeding. Cassava stem cuttings of similar lengths (about 20 cm each), were planted simultaneously in June 17, 2017 at both locations. Plants in each location were grown in a randomized block design consisting of 6 blocks each with the eight genotypes randomly assigned as plots. Each plot contained 8 plants grown in a 2x4 grid at 1m x 1m spacing. Due to plant-to-plant variation in sprouting, seedling and mid-season cessation of growth in some plants, survival tended to be higher in Ibadan than in Ubiaja (S1 Fig). Eight genotypes were selected from the IITA diversity population named the Genetic Gain Population. These genotypes were selected based on previous information about their flowering times (inflorescence initiation at the apical meristem) [25]. In cassava, inflorescence initiation (flowering) is associated with fork-type branching as explained in the Introduction, and by Perera et al. [10]. A high frequency of initiated inflorescences and their associated flower primordia abort before mature flowers are formed. Three categories were selected for our study, namely (i) early flowering (< 60 days after planting [DAP]), represented by IITA-TMS-IBA010615 and IITA-TMS-IBA020516, (ii) middle (60–99 DAP), represented by IITA-TMS-IBA030275, IITA-TMS-IBA010085, and IITA-TMS-IBA980002, and (iii) late (> 100 DAP), represented by IITA-TMS-IBA8902195, IITA-TMS-IBA000350, and TMEB419. They are available from the IITA germplasm bank (Ibadan, Nigeria; accession list: https://www.cassavabase.org/accession_usage). In this manuscript, these genotypes will be referred to as ‘615, ‘516, ‘275, ‘085, ‘0002, ‘2195, ‘350, and ‘419, respectively.

#### (b) Growth chamber

To transfer germplasm from Nigeria to our growth chamber facilities at Cornell University, one early genotype, IITA-TMS-IBA020516, and two late genotypes, IITA-TMS-IBA8902195 and IITA-TMS-IBA000350, were grown in tissue culture at the Genetic Resources Center, International Institute of Tropical Agriculture, Nigeria, and plantlets were screened to ensure absence of infection and other appropriate phytosanitary conditions. Tissue culture plants were shipped to Cornell University, Ithaca, NY, USA where they were transplanted to soil and grown several months to form plants with stems >15 mm diameter. Stakes of about 15 cm length were cut from the stems of established plants and used as propagules for experiments. Plants were grown in three growth chambers set at 22°C/18°C, 28°C/24°C, and 34°C/30°C, day/ night temperatures, respectively. Photoperiod was held constant at 12 h light and 12 h dark. Plants were completely randomized in each growth chamber. Each chamber had two replicates of each genotype. Two independent batches of this experiment were carried out for a total of four biological replicates. Growth chambers were Conviron Controlled Environments, Ltd (Winnipeg, Manitoba, Canada) model PGW 36 walk-in growth rooms (135 X 245 X 180 cm [ht.]) with ten 400 W high pressure sodium and ten 400 W metal halide lamps providing about 600 μmol photons (400–700 nm) m^-2^ s^-1^ at 1 m above the soil surface. Root-zone potting mix and fertilization were as previously described [11].

### Data collection

At Ubiaja and Ibadan, temperature was logged by Onset Computer, HOBO Pendant^®^ (https://www.onsetcomp.com/products/data-loggers/mx2202, Bourne, MA, USA) devices placed in ventilated reflective shelters [26] at 1.1 m height. Rainfall was logged by an automated tipping bucket rain gauge–RainWise^®^ (https://rainwise.com/rainlogger-complete-system, Trenton, ME, USA). Plants were examined weekly and flowering time was recorded as the days after planting (DAP) when the first reproductive branching (forking) occurred. In cassava, inflorescence initiation (flowering) is associated with fork-type branching as explained in the Introduction, and by Perera et al. [10]. Number of nodes was counted from the soil surface to the first fork on each plant. Plant height, whole plant fresh weight, storage root fresh weight and number of storage roots were recorded at 7 months after planting in the field and growth chamber. Data was collected using Field Book software application [27].

### Statistical analyses

Field data were modelled using a linear mixed model while growth chamber data was modelled using a simple linear model. In the field study, locations and genotypes were fixed effects, while blocks were random effects. In the growth chamber study, temperature (T), genotype (G), and T × G interaction were the modelled sources of variation. Both models were tested by analysis of variance. Flowering time and fraction of plants flowered were subjected to survival analysis using the Kaplan-Meier’s curve [28]. Multiple means comparison was conducted in the emmeans package [29] using the Tukey-HSD method. All analyses were conducted in R version 3.6.0 [30].

### Transcriptome analysis

Genotypes ‘0002 and ‘419 were selected for transcriptomic analysis in field grown plants while genotypes ‘516, ‘350, and ‘2195 were selected for analysis in controlled temperature environments. These genotypes represented the range of early and late flowering lines with varying degrees of environmental responsiveness. Leaf tissue samples were collected from the youngest fully expanded leaf on each plant. Three biological replicates were collected from field and growth chamber plants. The field samples were collected at 33 (Ibadan) or 36 (Ubiaja) DAP (preflowering) and 7d post flowering (relative to genotype development). In the growth chamber, samples were collected at 47 and 96 DAP. Samples were obtained in the late afternoon (Ubiaja and Ibadan) or within 1.5 h of the end-of-light period (growth chambers) and immediately placed in porous polyester tea bags and immersed in liquid N_2_ to freeze and for storage.

Total RNA was extracted from each sample by a modified CTAB protocol. For field samples about 0.2g of frozen leaf tissue were ground with mortar and pestle after which it was transferred to 1.5 mL Eppendorf tubes to which 1 mL of preheated (65°C) CTAB extraction buffer was added (Buffer comprised of 2% [w/v] CTAB detergent, autoclaved 0.1M Tris-HCl pH 8, 20mM EDTA, 1.4M NaCl and 2% PVP, with pH adjusted to 8.0). Samples were warmed at 65°C for 15 min with vortexing at 5-min intervals after which they were centrifuged at maximum speed for 5 min. To 1 mL of supernatant in a fresh Eppendorf tube, 1 mL of chloroform isoamyl alcohol (24:1) was added, vortexed and centrifuged for 10 min. Supernatant was collected in a clean Eppendorf tube to which cold 2-propanol was added (0.6 volume of supernatant) and mixed by inverting gently. Samples were centrifuged for 10 min at maximum speed to collect pellets which were washed in 70% ethanol and air dried. Pellets were redissolved in RNase free water, treated with DNase I and cleaned with RNA Clean and Concentrator (Zymo Research Corp., Irvine, CA, USA). RNA quality was determined by gel electrophoresis and RNA was bound to matrix in RNAstable (Biomātrica, San Diego, CA, USA) and shipped to Cornell University, Ithaca, NY, USA. At the destination, RNase free water was added to RNAstable to recover RNA for downstream assay as described below. Growth chamber samples were ground to a fine powder in a mortar and pestle chilled with liquid N_2_; about 0.5 g of the powder was vigorously mixed for 5 min with 1 mL of CTAB extraction buffer; 0.2 mL of chloroform was added and mixed for 15 s, tubes were centrifuged at 14,000 g for 10 min and the top layer was removed to a new tube. To each of these samples was added 700 μL of Guanidine Buffer (4M guanidine thiocyanate, 10 mM MOPS, pH 6.7) and 500 μL of ethanol (100%). This mixture was applied to a silica RNA column (RNA mini spin column, Epoch Life Science, Missouri City, TX, USA), then alternately centrifuged and washed with 750 μL of 1) Tris-ethanol buffer (10 mM Tris-HCl [pH 7.6], 1 mM EDTA, containing 80% [v/v] ethanol), 2) 80% ethanol (twice), and 3) 15 μL RNAase-free water (to elute the RNA). The RNA quality of field and growth chamber samples was evaluated for quality with an electrophoresis system (TapeStation 2200, Agilent Technologies, Santa Clara, CA, USA). Other downstream assays were same for both field and growth chamber samples.

cDNA libraries were prepared using the Lexogen Quantseq FWD kit [31] and DNA was sequenced by the 3’ RNASeq method [32] using an Illumina NextSeq500 sequencer at the Genomics Facility, Cornell Institute for Biotechnology. Trimmomatic and BBDuk software (https://sourceforge.net/projects/bbmap/; version 37.50; accessed 2020.01.21) was used to remove Illumina adapters, poly-A tails, and poly-G stretches, keeping at least 18 bases in length after trimming [33]. The trimmed reads were aligned to the *Manihot esculenta* genome assembly 520_v7 using the STAR aligner (version 2.7.0f) [34]. The number of reads overlapping each gene on the forward strand were counted using HTSeq-count (version 0.6.1; [35]).

Differential gene expression analysis was conducted using the DESeq2 package by Bioconductor [36]. Each transcript was annotated by the best match between *Manihot esculenta* genome v7 and the Arabidopsis genome as presented at Phytozome13 [37].

Gene ontology and enrichment analysis were carried out using the ShinyGO app (http://bioinformatics.sdstate.edu/go/) [38]. A combined list of Arabidopsis flowering genes were obtained from the Max Planck Institute (https://www.mpipz.mpg.de/14637/ Arabidopsis_flowering_genes) and Flowering Interactive Database (FLOR-ID) (http://www.phytosystems.ulg.ac.be/florid/) [19] and a list of hormone signaling genes sourced through the Database for Annotation, Visualization and Integrated Discovery (DAVID) (https://david.ncifcrf.gov/) [39] were used to examine the expression profiles of flowering and hormone signaling genes.

## Results

### Field experiment

#### Weather

**Fig 1:**
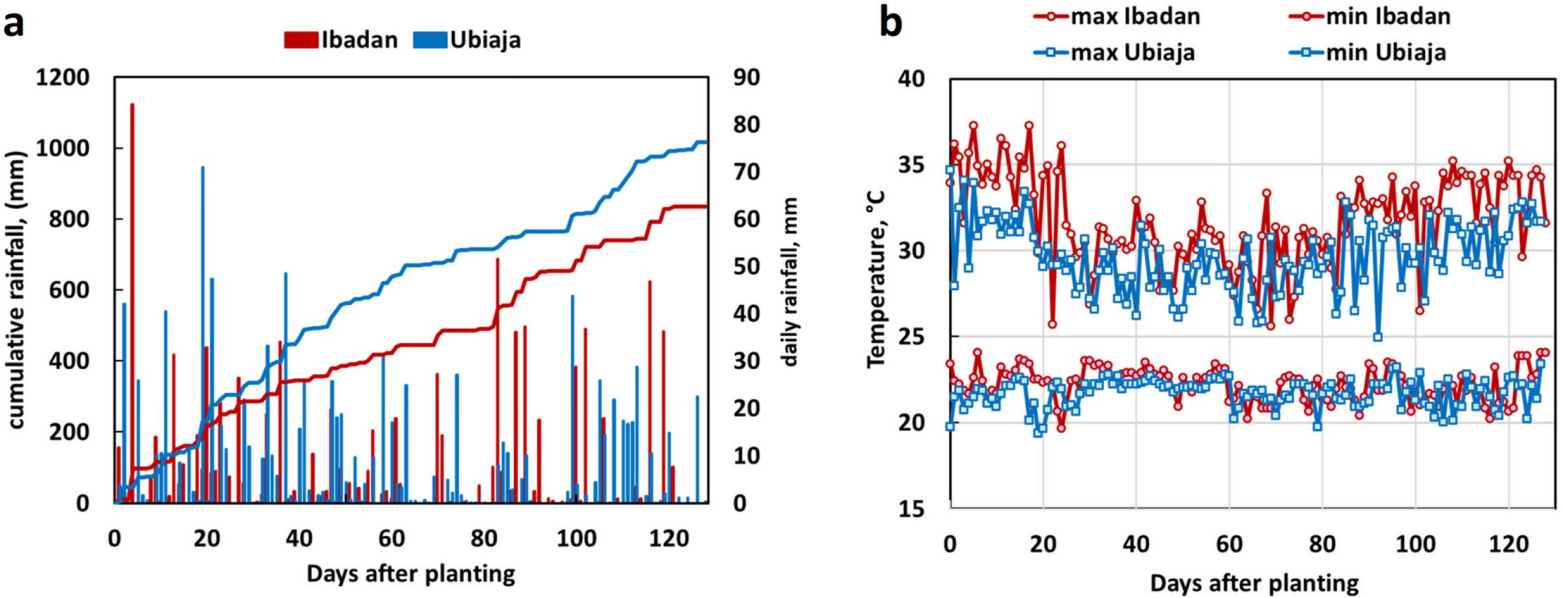
Weather data at field sites near Ubiaja (blue) and Ibadan (red), Nigeria. a) Cumulative rainfall (line graph, left axis) and daily rainfall events (bar graph, right axis). b) Maximum and minimum daily temperatures. Data are shown during the time of flowering from 0 to 130 days after planting (DAP) with respect to planting date of June 17, 2017.

Weather data collected from field sites in Ibadan and Ubiaja are shown in Fig 1. Cumulative rainfall at the two sites were similar in the first month, then diverged for the next two months with Ubiaja receiving more rainfall than Ibadan (Fig 1A). Temperature was measured in accordance with standard meteorological protocols [26] in shaded and ventilated shelters which housed the weather instrumentation. While nighttime temperatures were essentially the same at the two sites, day-time temperatures, as indicated by daily maxima, were generally cooler in Ubiaja than Ibadan (Fig 1B). During the time-frame before flower appearance (0 to 21 DAP), Ubiaja daily maximums averaged 3°C cooler than Ibadan (31.6 vs 34.6°C, respectively). At later time-frames, temperature differentials between Ubiaja and Ibadan were less: averages at Ubiaja and Ibadan, respectively, were 28.5 vs 30.1°C (Δ1.6°C) at 22–80 DAP, and 30.3 vs 32.7°C (Δ2.4°C) at 81–128 DAP.

#### Vegetative growth patterns under field environments of Ibadan and Ubiaja

**Fig 2:**
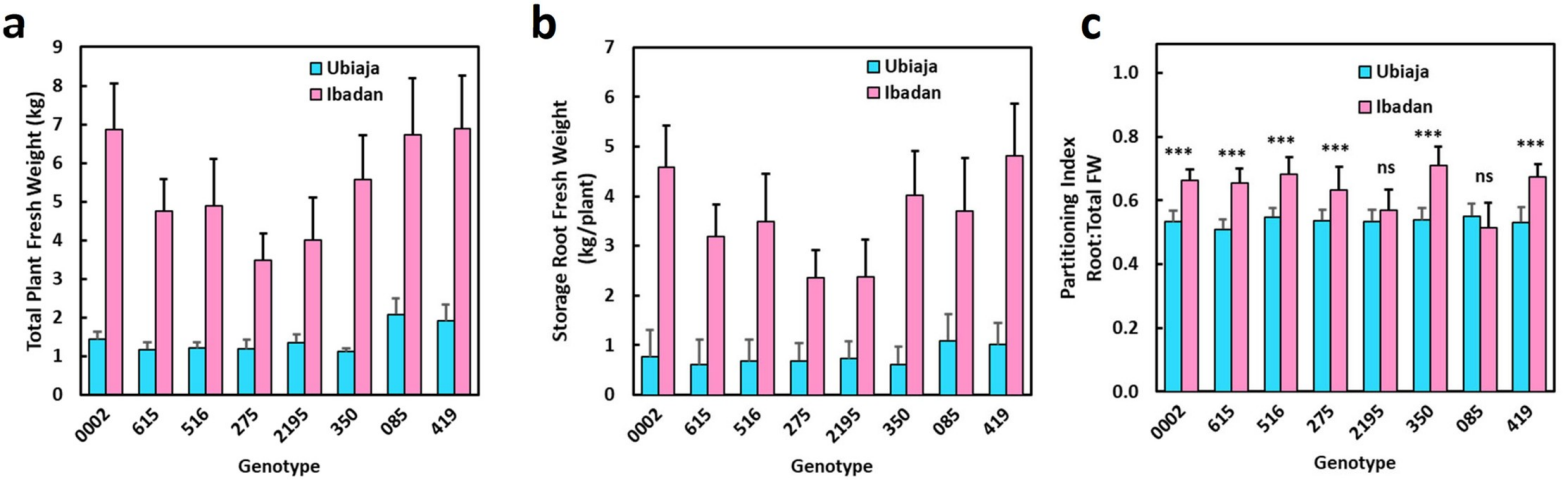
Vegetative growth at six months after planting of eight genotypes in the field experiment at Ubiaja (blue bars) and Ibaden (red bars). a) Total plant fresh weight (above-ground and storage roots); b) storage root fresh weight; c) partitioning index (storage root fresh weight/total plant fresh weight). All pairwise comparisons between locations were statistically significant (p≤0.001) for data in panels a and b. In panel c, comparisons that were not significant (ns) or were significant at p≤0.001 (***) are labelled. Data for mean partitioning index are reported though data were statistically analyzed on third order transformed (cubed) data.

For all eight genotypes, plants grown in Ibadan were larger than plants grown in Ubiaja (Fig 2A). Storage root fresh weight was also greater in Ibadan (Fig 2B). Similar trends were found in plant height, shoot weight, and number of storage roots (S2 Fig). While both above-ground and below-ground growth was greater in Ibadan than Ubiaja, the partitioning index (i.e. storage root weight/total plant weight on a fresh weight basis) was only about 20% higher in Ibadan than Ubiaja for most genotypes (Fig 2D). The pattern of vegetative growth between field locations indicated that plants were generally larger and more vigorous in Ibadan.

#### Flowering phenotype under field environments of Ibadan and Ubiaja

**Fig 3:**
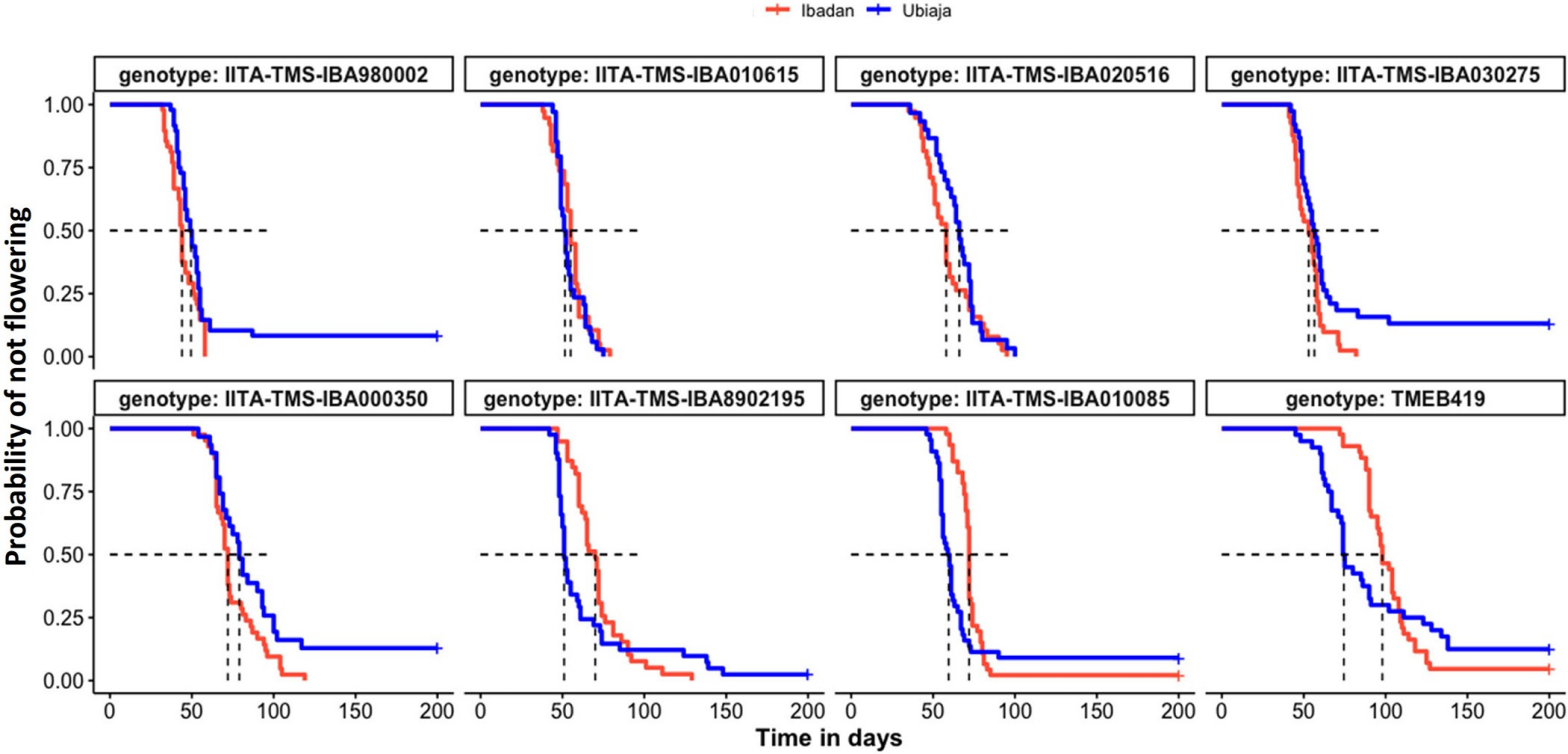
Flowering time probabilities for eight genotypes in Ubiaja and Ibadan. Kaplan-Meier curves show the time course of probability of not flowering in each location. Horizontal and vertical dashed lines indicate, respectively, 50% probability of flowering and corresponding days after planting (DAP).

In Fig 3, we plotted Kaplan Meier curves showing the probability of flowering (or the decline in the probability of not flowering) as a function of time. As described in the Materials and methods, flowering was defined as inflorescence initiation at the apical meristem, which is associated with fork-type branching. Included are both plants that flowered within the experimental period as well as plants surviving to the end of the experiment that did not flower. Hence, for some genotypes the probability of not flowering did not decline completely to zero. In Ibadan, most of the plants eventually flowered; however, in Ubiaja for ‘0002, ‘275, ‘350 and ‘419 between 10 and 15% of the lines failed to flower during the period of observation (up to 200 dap). This phenomenon resulted in cross overs of the flowering curves late in the season.

**Fig 4:**
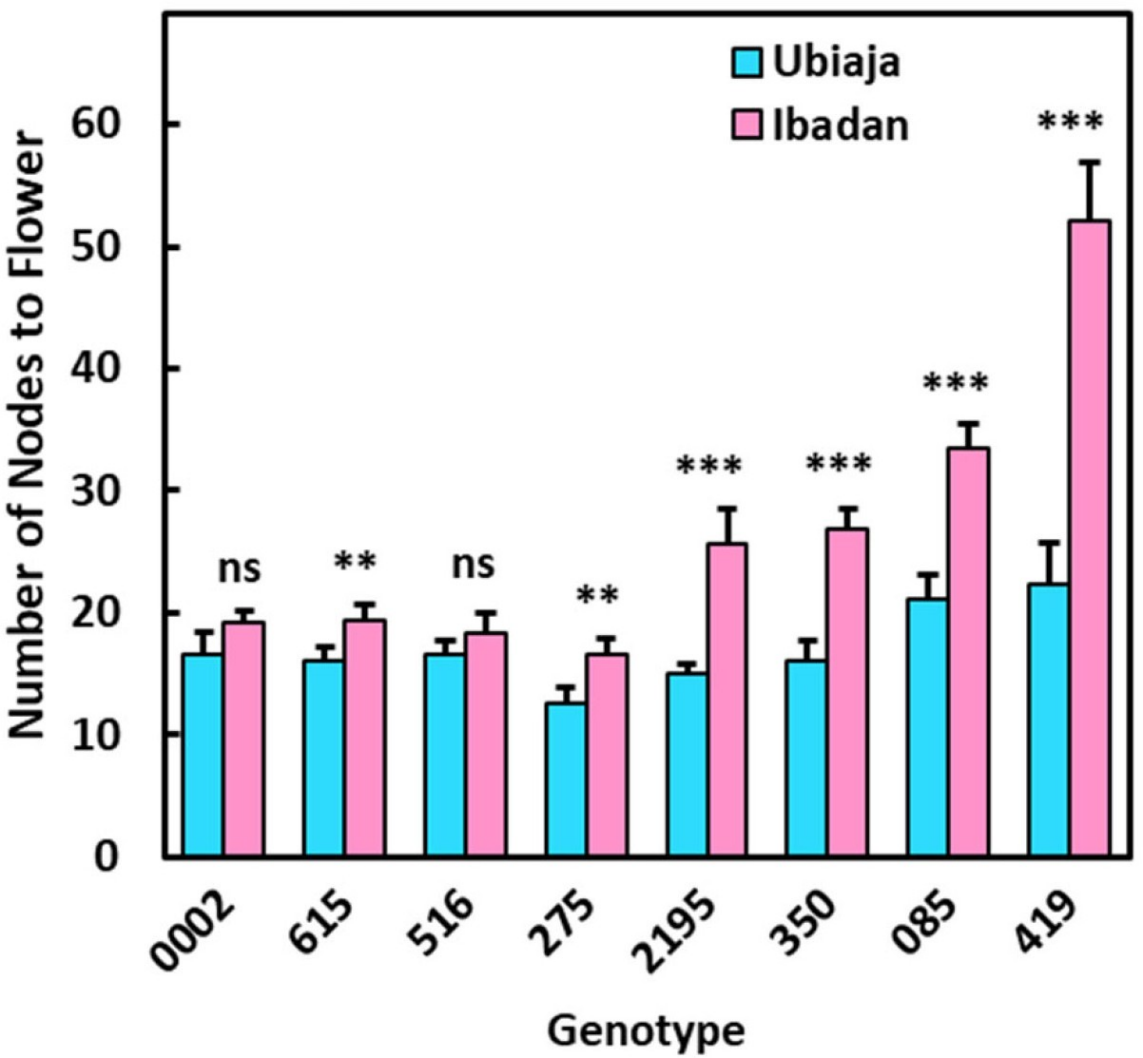
Number of nodes to flowering for eight genotypes grown in field locations of Ubiaja (blue) and Ibadan (red). Pairwise comparisons between locations are shown for each genotype at 0.05, 0.01 and 0.001 significance levels (*, **, ***), respectively, and those not significant (ns). Mean number of nodes are reported while data was square-root transformed for statistical analysis.

The extent to which time-to-flowering differed between the two locations was genotype-dependent. Genotypes ‘2195, ‘085, and ‘419 were earlier in Ubiaja, whereas ‘0002, ‘615, and ‘275 reached 50% flowering (half of the plants representing the genotype flowering) at nearly identical dates in the two locations. Genotypes ‘516 and ‘350 were unique as flowering was slightly (but not significantly) later in Ubiaja than Ibadan by chronological age (DAP), though the failure of these genotypes to reach 100% flowering in Ubiaja might have distorted the curves and average days to flower (S3 Fig). To provide another measure of the developmental time until flowering, we counted the number of nodes from the soil surface to the node at which flowering was initiated (fork). This approach recognizes that warmer temperature can have a general hastening effect on many developmental processes including growth, leaf and node production while our interest is on potential opposing inhibitory effects of warmer temperatures on flower initiation. These data revealed that half of the genotypes (‘0002, ‘615, ‘516, and ‘275) flowered at Ubiaja and Ibadan within just a few nodes of each other (Fig 4). In contrast, the other group of genotypes (‘2195, ‘350, ‘085, and ‘419) flowered with much greater nodes-to-flower in Ibadan than Ubiaja. The genotypes ‘2195, ‘350, and ‘085 were delayed developmentally such that in Ibadan (compared to Ubiaja) they developed 60–70% more nodes before they flowered, and ‘419 developed 133% more nodes before it flowered. It is noteworthy that whereas genotypes could be grouped into two categories based on the extent to which flowering was delayed on the basis of nodes-to-flowering in Ibadan, all genotypes responded similarly to environment for their vegetative growth and root partitioning index in Ubiaja and Ibadan (Fig 2C).

### Controlled temperature experiment with contrasting genotypes

**Fig 5:**
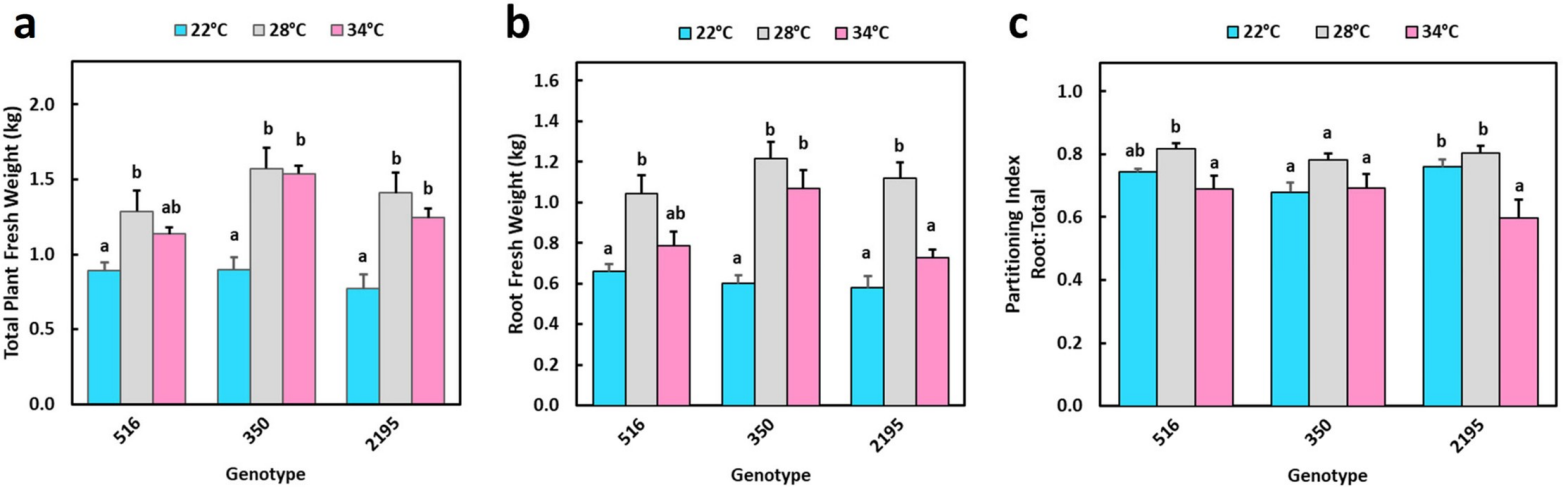
Vegetative growth of three genotypes under controlled temperatures. a) Total plant fresh weight; b) storage root fresh weight; c) partitioning index (storage root fresh weight/total plant fresh weight). Temperature treatments within each genotype which have different lowercase letters are significantly (p≤0.05) different using Tukey’s HSD test. Comparisons and letter assignments were based on estimated marginal means (EMMs, least-squares means), as appropriate for statistical comparisons; arithmetic means and SEMs are plotted.

Given that field locations differed in day-time temperature (Fig 1B), we evaluated genotypes for their flowering response to three temperatures in controlled-environment growth chambers. The genotypes selected for this experiment represented the range of response: ‘350 and ‘2195, for which flowering was delayed in the warmer environment of Ibadan such that it had more nodes-to-flower in Ibadan than Ubiaja, and ‘516, which flowered after approximately the same number of nodes in both environments. Growth of total plant fresh weight was greater at 28°C than 22°C for all three genotypes, and plateaued at 34°C (Fig 5A). Root fresh weight also increased from 22°C to 28°C in all three genotypes, but tended to decrease in ‘516 and ‘2195 from 28°C to 34°C (Fig 5B). Similar temperature trends were observed in above-ground shoot growth and plant height (S4 Fig). Given the similarity of temperature response in shoots and roots, the root partitioning index (root weight:total plant weight) was the same at 22°C and 28°C for all three genotypes, and decreased only modestly from 28°C to 34°C (Fig 5C). Warmer temperatures in the growth chamber tended to decrease the number of storage roots per plant (S4 Fig).

**Fig 6:**
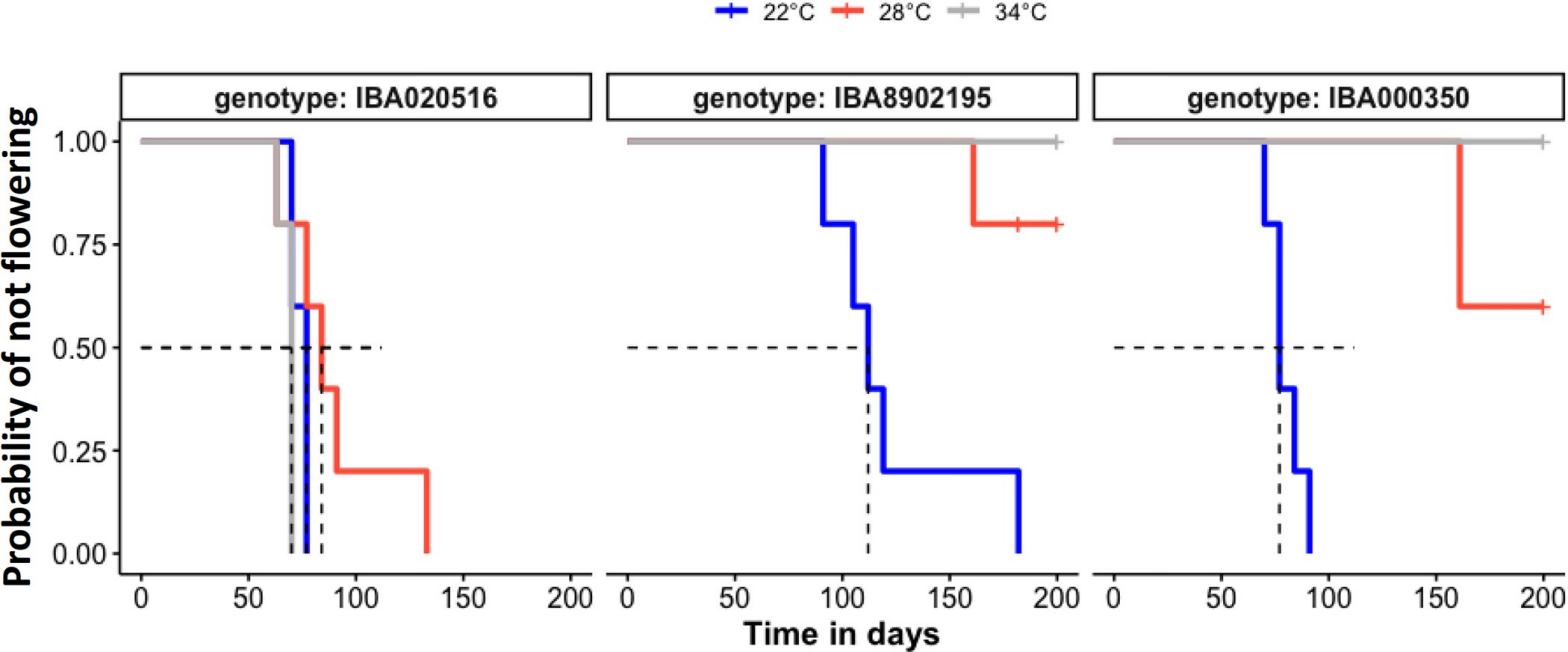
Flowering time probabilities for three genotypes in controlled-environment growth chambers at three temperatures treatments (day/night): 22/18°C (blue), 28/22°C (red), and 34/30°C (gray). Kaplan-Meier curves show the time course of probability of not flowering in each location. Horizontal and vertical dashed lines indicate, respectively, 50% probability of flowering and corresponding days after planting (DAP).

**Fig 7:**
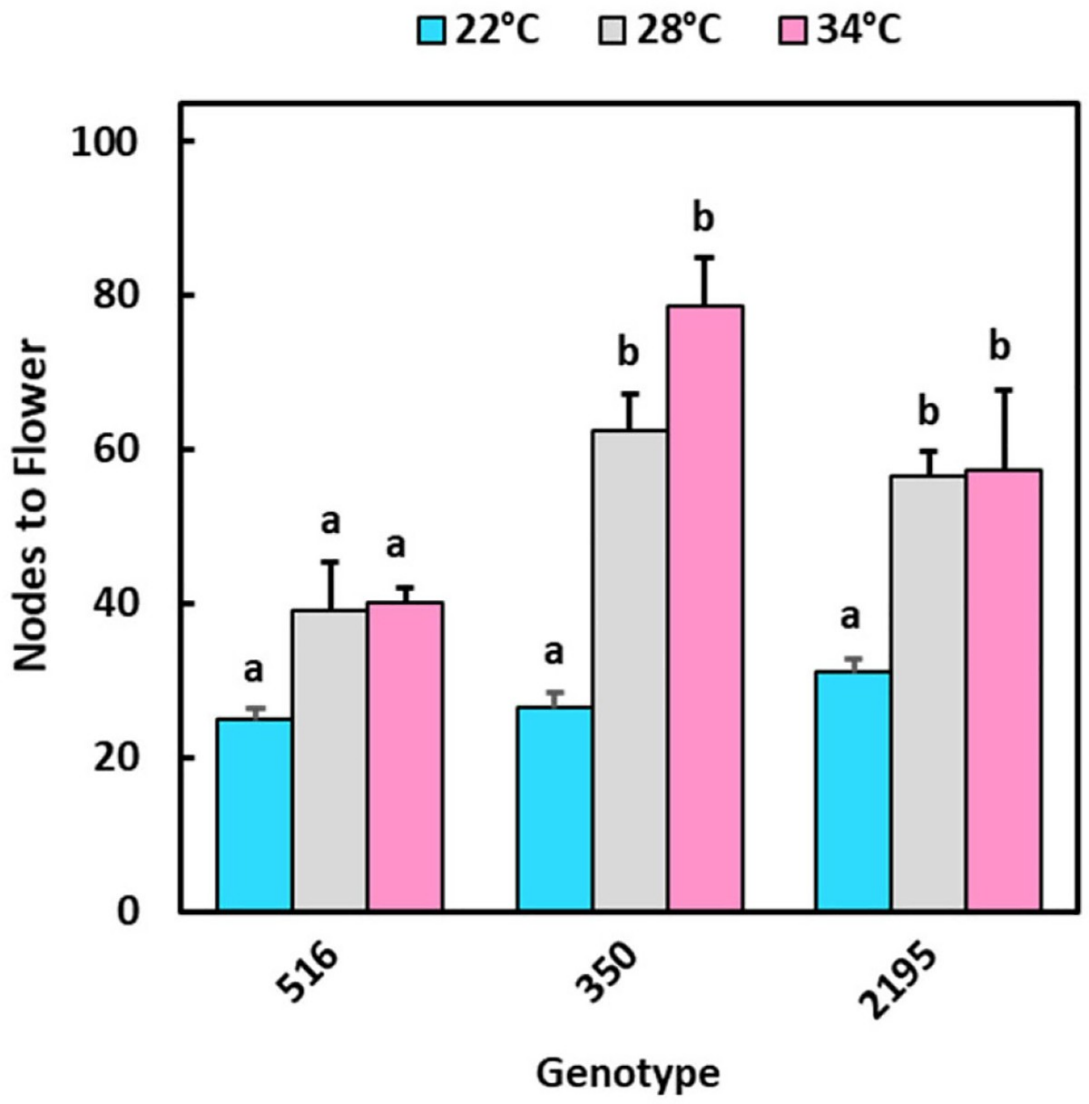
Duration of plant development before flowering in controlled-environment growth chambers at three temperatures treatments (day/night): 22/18°C (blue), 28/22°C (gray), and 34/30°C (red). Number of nodes on main stem to last node before flowering at the apex, or maximum countable nodes in cases where no flowering occurred within the period of observation (200 d). Temperature treatments within each genotype with different lowercase letters are significantly (p≤0.05) different using Tukey’s HSD test. Comparisons and letter assignments were based on estimated marginal means (EMMs, least-squares means), as appropriate for statistical comparisons; arithmetic means and SEMs are plotted.

To investigate the effect of temperature on earliness of flowering, we used data from the growth chamber experiment and modelled the probability of flowering (or the decline in the probability of not flowering) as a function of time using the Kaplan Meier method (Fig 6). For this, we used the observed flowering time where flowering occurred within the duration of experiment, or for plants that did not attain flowering during the period of observation, the maximum duration of experiment. At 22°C, for all genotypes, 100% of the plants flowered during the experiment. In contrast, at warmer temperatures (28 and 34°C) only genotype ‘516 attained 100% flowering, and its flowering was only slightly delayed at 28°C (Fig 6, left panel). Genotypes ‘350 and ‘2195, however, flowered poorly at warmer temperatures–flowering was completely absent at 34°C, while flowering was attained in only 20 to 30% of the plants at 28°C within the period of experiment (Fig 6, two right panels). Similar trends were observed when the data was expressed as average age to flowering (S5 Fig). The number of nodes to flowering, an alternative measure of developmental timing, confirmed the genotypic differences in temperature responsiveness (Fig 7). The number of nodes to flowering in ‘516 did not differ statistically (p≤0.05) amongst temperatures, confirming this genotype’s relative insensitivity to a delaying effect by warm temperatures. This finding corresponds with ‘516’s insensitivity in number of nodes-to-flowering among the different environments of Ibadan and Ubiaja (Fig 4). In contrast, nodes-to-flowering (or maximum number of nodes countable) as an index of development, indicated that flowering in ‘350 and ‘2195 was substantially delayed at warmer temperatures. These genotypes flowered after significantly (p≤0.01) fewer nodes at 22°C than at 28°C and 34°C where flowering was partial or completely absent (Fig 7).

### Differential gene expression analysis

Transcriptomes were analyzed in mature leaves of plants grown in the two studies (Field Environments and Growth Chamber) with respect to the following variables: 1) Environment (Ubiaja versus Ibadan in the field study; three temperatures in the growth chamber study); 2) Stage of plant development relative to flowering, where the early stage was before flowering and the later stage was post flower appearance, and 3) Genotype, where lines were chosen to represent a range of environmental responsiveness and earliness of flowering.

#### Field study of differentially expressed genes

**Fig 8:**
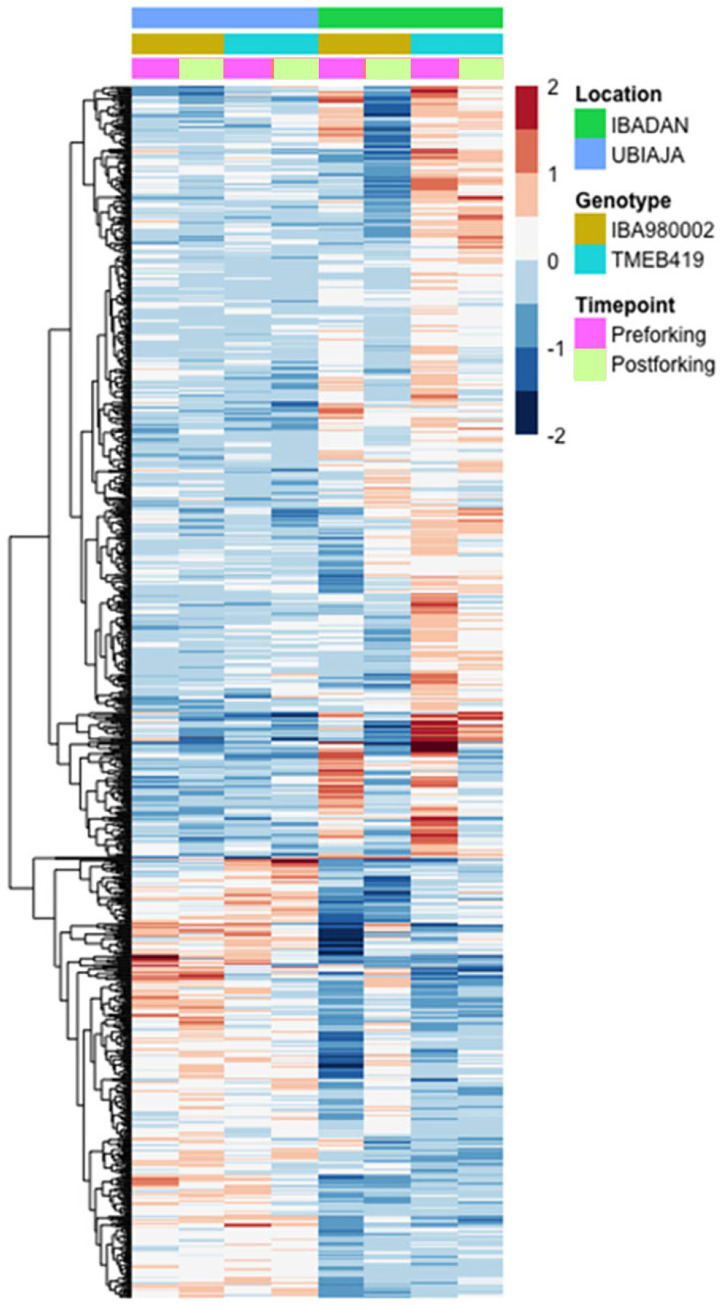
Relative expression (log_2_ scale) of differentially expressed genes in response to field location (Ibadan vs Ubiaja), genotype (‘0002 vs ‘419), and timepoint of development (pre-flowering vs post-flowering). Shown are 1074 genes with significant (p_adj_≤0.05) differential expression among averages of biological replicates per time point, genotype and location. Color scale indicates log_2_ fold changes relative to the geometric mean of all samples for each gene. Legend in header indicates color coding of variables. Full data set is in S2 Table.

Under field conditions, for the combined genotypes and sampling dates, 1074 genes were differentially expressed (p_adj_≤0.05, p-value adjusted for multiple comparisons by Benjamini-Hochberg) between Ubiaja and Ibadan. In comparison with expression in Ibadan, 390 genes had higher expression in Ubiaja while 684 genes had lower expression in Ubiaja (Fig 8) (S1 Table). Expression tended to respond to location (Ibadan vs Ubiaja) to a greater extent at the pre-flowering than post-flowering stage of development. In the ‘0002 genotype, there were 823 genes that changed 2-fold (doubling or one half) or more in Ibadan vs Ubiaja, pre-flowering but only 229 post-flowering; in ‘419 there were 855 pre-flowering and 577 post-flowering. The genotype ‘419, which was more responsive to location in its timing of flowering (Fig 7), also had more genes post-flowering that changed by log_2_ of |≥2| than ‘0002: 136 and 11 genes, respectively (S2 Table).

Enrichment analysis indicated that the categories of genes that were significantly overrepresented among the genes that had higher expression in Ubiaja than Ibadan were several that relate to responses to environmental factors, including “Response to abiotic stimulus” (54 genes, p = 9.5E-5) and “Response to oxidation-reduction process” (45 genes, p = 1.3E-3) (S3 Table). Overrepresented categories among genes with lower expression in Ubiaja than Ibadan included “Cell wall organization or biogenesis” (57 genes, p = 1.2E-13) and “Polysaccharide metabolic process” (43 genes 2.6E-12).

**Fig 9:**
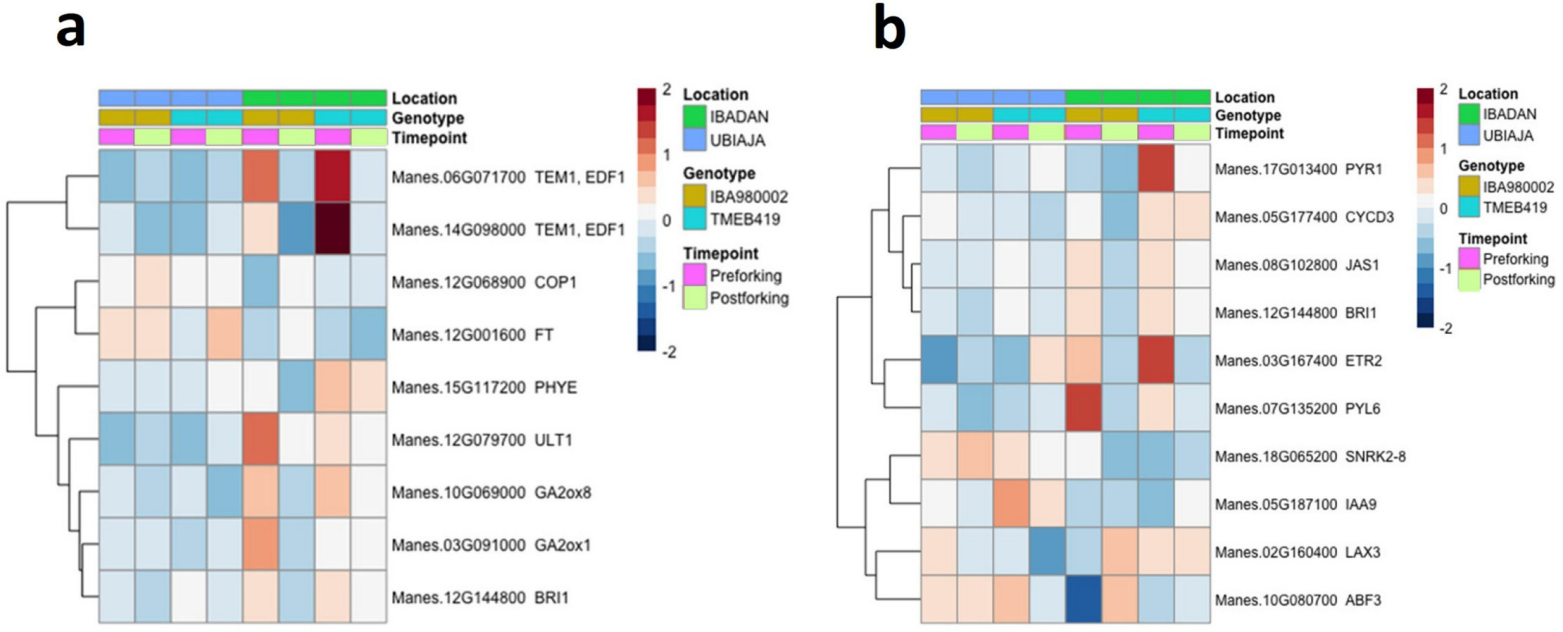
Flowering time and hormone signaling genes differentially expressed in leaves of two genotypes at two timepoints in field locations of Ubiaja and Ibadan. The heat map shows relative expression (log_2_ scale) across location (Ubiaja vs Ibadan), genotype (‘0002 vs ‘419), and timepoint of development (preflowering and postflowering). **a**) Flowering time genes; **b**) Hormone signaling genes. Each gene is identified by its *Manihot esculenta* number (Manes) and its closest Arabidopsis homolog. Legend in header indicates color coding of variables. The indicated cassava homolog of Arabidopsis *FT*, Manes.12G001600, has been named *MeFT1* [11].

Although the leaf transcriptome in this study is likely to have numerous differentially expressed genes among the tested environments for factors that relate to leaf stress, photosynthesis, and leaf metabolic processes, we focused our analysis on genes related to flowering and related signaling. From a list of 240 flowering genes (see Materials and methods), nine were differentially expressed in the field transcriptome (Fig 9A). These genes generally responded to location (Ubiaja vs Ibadan) at the pre-flowering timepoint, with some of them having higher expression in Ibadan (eg. two *TEM1* homologs), whereas *MeFT1*, a cassava homolog of *FT*, had higher expression in Ubiaja than Ibadan.

Among a list of 160 hormone signaling genes, in the signaling pathways of eight plant hormones (see Materials and methods), 10 hormone signaling genes were differentially expressed in the field-grown plants (Fig 9B). These genes were involved in signaling or response to abscisic acid (4 genes: *PYR1*, *PYL6*, *SNRK2-8*, *ABF3*), auxin (2 genes: *IAA9*, *LAX3*), cytokinin (*CYCD3*), ethylene (*ETR2*), jasmonic acid (*JAS1*) and brassinosteroid signaling (*BRI1*). While the patterns of expression for these genes varied, several exhibited differential expression between the Ibadan and Ubiaja locations at the pre-flowering stage. For example, at the pre-flowering stage, homologs of *PYR1* and *ETR2* had higher expression in the poor-flowering line ‘419 than in ‘0002, and in the poor-flowering location Ibadan than in Ubiaja.

#### Controlled temperature study of differentially expressed genes

**Fig 10:**
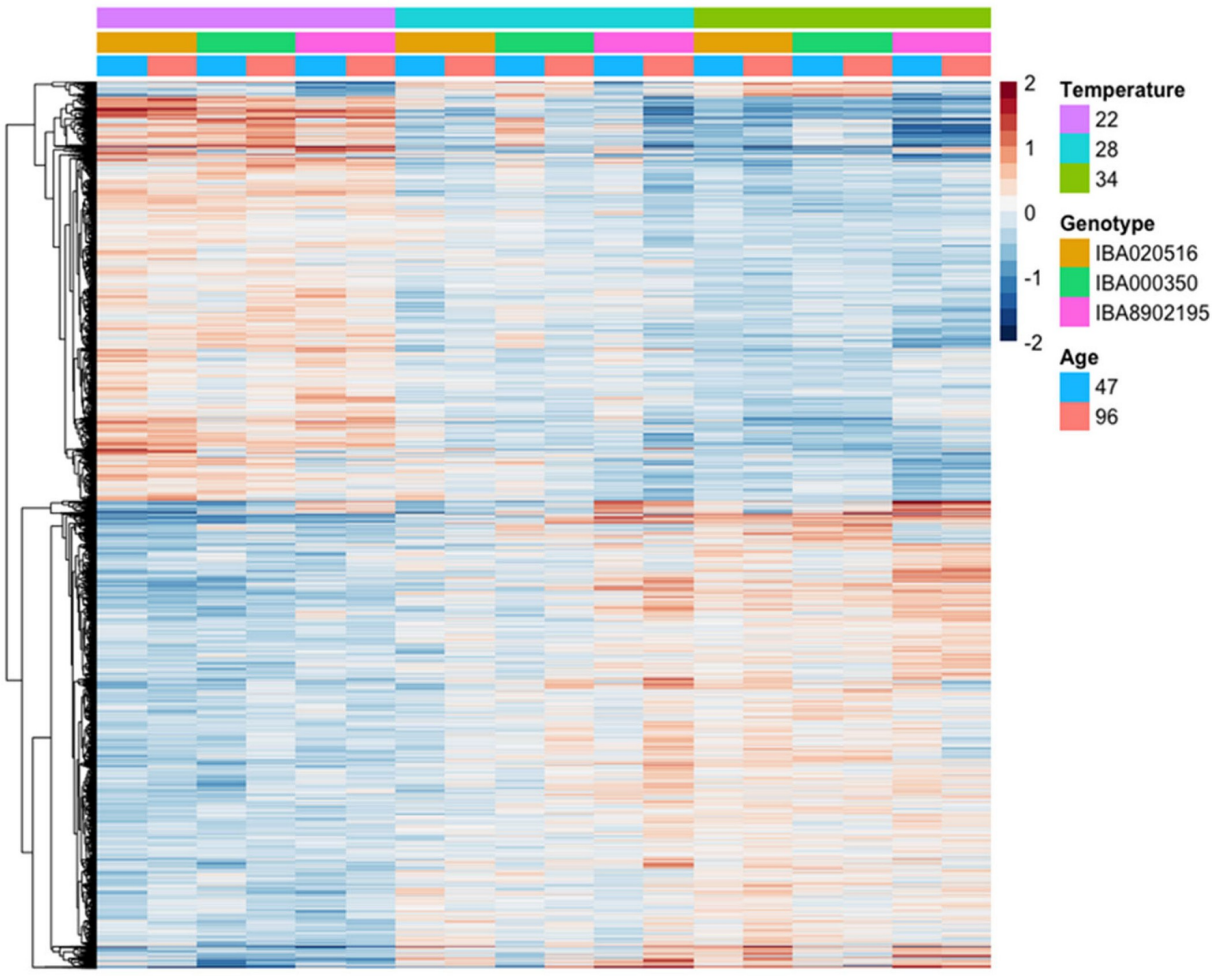
Relative expression (log_2_ scale) of differentially expressed genes in response to controlled-environment temperature (22, 28 or 34°C), genotype (‘516, ‘350, ‘2195), and timepoint of development (47 and 96 DAP). The figure shows 7253 genes differentially expressed (p_adj_≤0.05) (averages of biological replicates per time point, genotype and location). Color scale indicates log_2_ fold changes. Legend in header indicates color coding of variables.

Our analysis of weather in Ubiaja and Ibadan indicated that day-time temperature in Ubiaja was cooler than Ibadan (Fig 1). We hypothesized that the cooler temperatures might be a factor influencing earlier flowering in Ubiaja, and that the genotypic differences in flowering (Fig 6) would relate to their transcriptomes. Under controlled conditions with 22°C as reference, 7253 genes were differentially expressed (p_adj_≤0.05) in response to the three temperatures studied– 3940 had higher expression and 3313 lower expression at warmer temperatures of 28 and 34°C (Fig 10; S4 and S5 Tables). For a large fraction of the genes, expression was distinctly greater (upper half of figure) or lower (lower half of figure) at 22°C compared to the other two temperatures, with expression at 28°C intermediate, and similar to that at 34°C. Enrichment analysis indicated that genes with higher expression at 28 and/or 34°C were over-represented in the gene ontology category “response to stress” (588 genes; p = 1.2E-17), whereas genes with lower expression at 28 and/or 34°C were over-represented in the category “small molecule metabolic process” (380 genes; p = 8.4E-35). The full data set of top-10 categories is in S6 Table.

**Fig 11:**
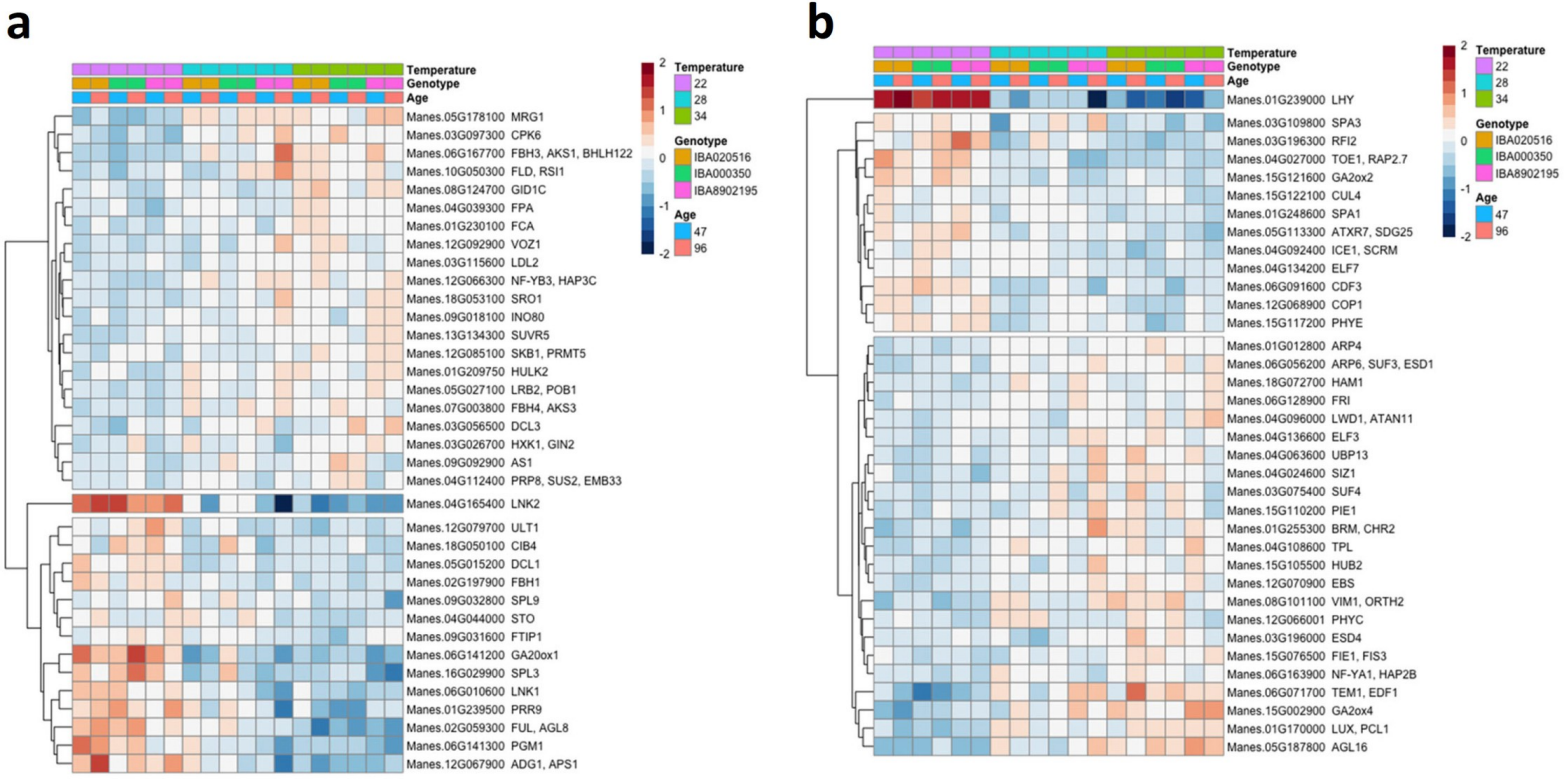
Relative expression (log_2_ scale) of differentially expressed flowering-time genes in response to controlled-environment temperature (22, 28 or 34°C), genotype (‘516, ‘350, ‘2195), and timepoint of development (47 and 96 DAP). a) genes which have a positive effect (hastening) on flowering time in Arabidopsis; b) genes which have a negative effect (delaying) on flowering time in Arabidopsis. Each gene is identified by its *Manihot esculenta* number (Manes) and its closest Arabidopsis homolog. Color scale indicates log_2_ fold changes. Legend in header indicates color-coding of variables.

Ninety-six known flowering-time genes were differentially expressed under controlled temperature, split nearly evenly between positive and negative effectors, 49 and 47 genes respectively (Fig 11). Among these genes, those known to enhance flowering (based on characterization in Arabidopsis) included *GA20ox1*, *SPL3*, *LNK1*, *PRR8*, *PGM1*, *FUL*, *ADG1* and *LNK2*. These had higher expression at 22°C than at warmer temperatures (28°C and 34°C) for both timepoints (47 and 96 DAP) (Fig 11A), consistent with our observation of earlier flowering at 22°C. Among genes which negatively influence flowering time in Arabidopsis, about 66% had lower expression at 22°C than at warmer temperatures, while 33% had higher expression under 22°C (Fig 11B).

**Fig 12:**
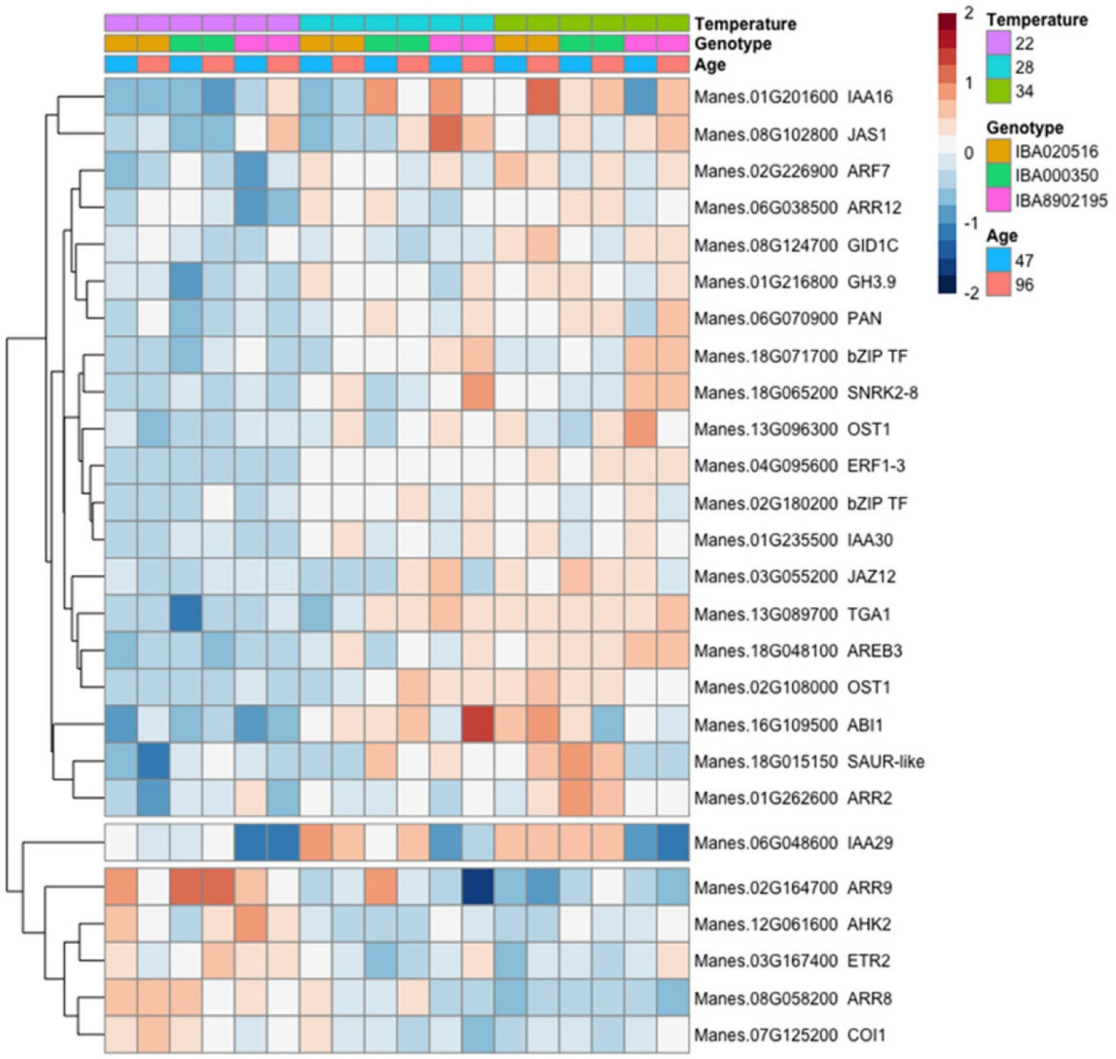
Relative expression (log_2_ scale) of differentially expressed hormone signaling genes in response to controlled-environment temperature (22, 28 or 34°C), genotype (‘516, ‘350, ‘2195), and timepoint of development (47 and 96 DAP). Each gene is identified by its *Manihot esculenta* number (Manes) and its closest Arabidopsis homolog. Color scale indicates log_2_ fold changes. Legend in header indicates color coding of variables.

Among hormone signaling genes (see Materials and methods for list of selected genes), most of those which were differentially expressed (p_adj_≤0.05) in response to temperature treatments had lower expression at 22°C and higher expression at warmer temperatures (Fig 12). Consistent with our field experiment, several genes involved in abscisic acid signaling or response (*OST1*, *ABI1*, *SNRK2-8*, *AREB3*) and auxin (*SAUR-like*, *IAA16*, *ARF7*, *IAA30*, *IAA29*, *GH3*.*9*) were more highly expressed in the higher temperature environments. In addition, other hormone signaling pathways associated with stress, such as jasmonic acid signaling genes (*JAS1*, *JAZ12*), GA receptor (*GID1C*), bzip transcription factors involved in multiple hormone signaling pathways (*TGA1*, *PAN*), and translation terminator *ERF1-3*, had lower expression at 22°C. In contrast, the negative regulator of ethylene stress-hormone pathway, ethylene receptor *ETR2*, had higher expression at 22°C. Cytokinin signaling was regulated in the direction of suppressed signaling at 22°C: the cytokinin receptor *AHK2* and A-type response regulators (*ARR8* and *ARR9*), which function as negative regulators of cytokinin signaling, had higher expression levels at 22°C, whereas B-type cytokinin response regulators (*ARR12* and *ARR2*) mediating cytokinin positive effects had lower expression levels at 22°C.

## Discussion

### Environmental factors that affect flowering

The current study and previously published work have indicated that flowering is earlier and more abundant in Ubiaja than Ibadan [23–25,40]. One goal of the present study was to provide insight on the underlying basis of this difference. In previous studies, it was hypothesized that differences in soil type or fertility might explain the better flowering in Ubiaja. Simwambana et al. [24] conducted studies that involved growing plants in large pots (160 kg) of soil that were reciprocally exchanged between Ibadan and Ubiaja. They concluded that soil type or fertility did not explain the considerably better flowering in Ubiaja. In the present study, plant growth was more vigorous in Ibadan than Ubiaja, as evidenced by plant height, storage root weight and numbers (Fig 2). Storage-root partitioning index was slightly higher in Ibadan (Fig 2). These data indicate that the Ibadan environment provided better vegetative growth and storage-root production, but was less favorable for flowering than Ubiaja. Weather data indicated that daytime temperatures were higher in Ibadan than Ubiaja, particularly in the pre-flowering period of the first three weeks after planting (Fig 1). In controlled-temperature studies, we also found that in comparison with 22°C, warmer temperatures of 28 and 34°C increased vegetative growth and storage root production, and that storage root partitioning index was not significantly affected by growth temperature (Fig 4). Furthermore, flowering was earlier in the cooler environment (22°C) than the warmer environments (28 and 34°C), and at these warmer temperatures, flowering did not occur within the experimental period in some genotypes (Fig 6 and 7), consistent with earlier flowering in the cool environment of Ubiaja (Fig 4). A possible alternative hypothesis for the earlier flowering in Ubiaja is that in this environment vegetative growth is suppressed and as a consequence this provides better photosynthate supply for sugar signaling and flower development [41]. However, total plant growth, which is an indication of whole-plant photosynthesis, was only about 25% as much in Ubiaja than Ibadan, which challenges this hypothesis. In some plant systems, flowering is evoked by a prior water deficit [42,43], and studies of cassava have shown a correlation between the dry season in mountainous areas and flowering [44]. However, in the present study, better flowering in Ubiaja was associated with more rainfall than in Ibadan (Fig 1). Furthermore, studies of water deficit in cassava have indicated that root partitioning index was not significantly affected by water unless water limitation was extreme and prolonged to the point of remobilization from stem and root storage reserves [45]. Thus, the available evidence indicates that temperature is a contributing factor to explain flowering differences in various environments.

#### Early flowering genotypes were relatively insensitive to the environment while late flowering genotypes were delayed by the Ibadan environment and warmer temperatures

In our field study, genotypes ‘0002, ‘275, ‘615 and ‘516 flowered early on the basis of their fewer nodes to flowering than the other four genotypes in the study (Fig 3). Furthermore, flowering rates for these genotypes were stable across Ubiaja and Ibadan environments with a difference of less than 5 nodes-to-flower between Ubiaja and Ibadan (Fig 4). Nodes-to-flower is widely used by researchers as a measure of development that has a similar advantage to the use of growing-degree-day (GDD) as a basis to express development in environments differing in temperature. Nodes-to-flower are units which factor out temperature dependence due to general growth processes. Studies on a number of plant systems have indicated that leaf initiation (to which node number is associated) is one of the developmental processes for which temperature dependence follows a GDD relationship [46]. By using nodes-to-flower, we are able to reveal other temperature-dependent factors (such as our current finding that in some genotypes flower initiation happens at more advanced stages of development in warm environments). We also tried using GDD on our data; however, we do not have prior estimates of the base temperature for cassava, and it seems that genotypes might differ from one another in such relationships. Similarly, under controlled environments with day-time temperatures from 22 to 34°C, one representative of this group of early, stable genotypes, ‘516, had similar nodes-to-flower at all three temperatures (Fig 7). In contrast, the genotypes ‘350, ‘2195, ‘085 and ‘419 showed a plastic response to environment with large differences in their flowering time between environments and much later nodes-to-flower in the warmer-temperature Ibadan environment than in Ubiaja. The difference between this group of genotypes and the stable group was in their much later flowering in Ibadan. Similarly, when two representatives genotypes from this plastic responding group, ‘350 and ‘2195, were grown in controlled temperatures, they had similar nodes-to-flower as ‘516 at 22°C, however, they were much later at warmer temperatures (28°C and 34°C) (Fig 7). These findings are in agreement with those of Adeyemo et al. [11], who showed that the early, stable genotype, ‘0002, flowers at about the same days-to-flower at 22 and 28°C, whereas the late-flowering plastic genotype ‘419 initiates flowers at 22°C but did not flower at 28°C. Such genotypic differences in the extent to which flowering is delayed by warmer temperatures have also been reported in other species such as the long-day-plant pea (*Pisum sativum*) [47], and many others [48].

A meta-analysis of flowering time data on more than 700 genotypes grown at Ubiaja and Ibadan over three seasons [25] showed that flowering time in both locations was between 60 and 70 DAP with a skew in Ibadan toward greater days-to-flower for some fraction of the genotypes (S6 Fig). It is therefore likely that the flowering times of early, stable genotypes represent the minimal flowering time of cassava in the absence of environmental conditions that have a delaying effect. Warmer temperatures may induce regulatory systems in plastically responding genotypes which in turn delay flowering. Our studies indicate that later genotypes primarily differ from early ones in the extent to which their flowering is delayed in unfavorable environments, i.e. Ibadan and warm growth chambers.

Several members of the Euphorbiaceae family, to which cassava belongs, are known to flower more readily at moderately cool temperatures than at warmer temperatures, including rubber tree (*Hevea brasiliensis*) [49], poinsettia (*Euphorbia pulcherrima*) [50], and leafy spurge (*Euphorbia esula*) [51]. Other tropical perennials are also known to be induced to flower by cool ambient temperature, notably Lychee (*Litchi chinensis*) and Mango (*Mangifera indica*). In Lychee, warm temperatures stimulate vegetative growth while cool temperatures of 20°C or less promote reproductive growth [52,53]. In mango, cool temperatures of 15°C stimulated flowering [43]. Furthermore, in mango, water stress at cool temperatures causes profuse flowering but water stress under warm temperatures did not induce flowers [43]. This stimulation of flower induction by cool temperature in the tropics has been suggested to be related to the drop in temperature preceding or coinciding with the onset of rains, thus serving as an environmental cue [42].

#### Flowering repressors were highly expressed in Ibadan before flowering

The current study determined the transcriptome of expressed genes in recently matured leaves of the Ibadan-Ubiaja field experiment, and of a temperature comparison in the growth chamber experiment. In the Ubiaja environment, which was favorable for flowering, cassava homologs of known Arabidopsis flowering repressors, including *GIBBERELLIC ACID 2 OXIDASE 1* (*GA2ox1*), *GIBBERELLIC ACID 2 OXIDASE 8* (*GA2ox8*), *TEMPRANILLO 1* (*TEM1*) and *PHYTOCHROME E* (*PHYE*) [19] generally had low expression levels before and after flowering (Fig 9). In contrast, these genes were highly expressed in the poor-flowering Ibadan environment before flowering, and their expression returned to baseline after flowering. On the other hand, a cassava homolog of the flower-inducing regulatory factor *FLOWERING LOCUS T* (*MeFT1*), was generally expressed pre-flowering at higher levels in Ubiaja than Ibadan (Fig 9). Adeyemo et al. [11] previously showed that *MeFT1* expression was related to flowering tendency, as it was expressed at higher levels in ‘0002 (early genotype) than in ‘419 (late, with a plastic phenotype). Studies of cassava in various locations in Vietnam have shown that in a mountainous region, flowering is stimulated during the dry season, and correspondingly, this is when expression of *MeFT1* is highest [44]. FT (florigen) has been established in angiosperms as a mobile long distance signaling protein that is transcribed and translated in leaves, and the protein moves from leaf to the shoot apical meristem where it induces flowering [20,54]. While our RNA-seq methodology did not have sufficient limit-of-detection to assess *MeFT2* in either of our studies, and *MeFT1* in the growth chamber study, in the field study, *MeFT1* was higher in Ubiaja than in Ibadan, consistent with better flowering in Ubiaja (Figs 4 and 9). In future studies, attention should be paid to both factors that promote flowering and those that suppress flowering. Flowering may require an optimal ratio between florigens and anti-florigens as has been demonstrated in tomato (*Solanum lycopersicum*), where flowering and plant architecture is determined by the local balance of florigenic and anti-florigenic signals in respective organs [55,56].

#### Flowering phenotype correlates with *TEM1* expression under field and controlled temperature conditions

A cassava homolog of the Arabidopsis flowering-time repressor *TEM1* (Manes.06G071700), had low expression levels under all the tested conditions in which cassava flowering was earlier (i.e. Ubiaja environment and 22°C) (Figs 4 and 7), whereas it was elevated where flowering was later (Ibadan; 28 or 34°C). In Arabidopsis, *TEM1* has a roles in maintaining juvenility [57] and responding to other environmental signals to regulate flowering time [58,59], and it directly represses *FT* expression under conditions that delay flowering [60]. There is also evidence that *TEM1* has a role in ambient temperature response in Arabidopsis, where flowering is repressed at cool temperatures [61], and correspondingly, *TEM1* expression is elevated [62]. The temperature response in cassava is consistent with this, although the direction of the temperature response in cassava is the reverse of that in Arabidopsis. In the current study, cassava flowering was repressed by warm temperatures (Fig 7), and *TEM1* expression was elevated in warm temperatures corresponding to a postulated role as a flowering repressor. Consistent with the current work, enhancement of cassava flower development by the anti-ethylene silver thiosulfate (STS) and the cytokinin benzyladenine downregulated *TEM1* in apical-region tissues [63]. Hence, the expression pattern of *TEM1* identifies it as a key candidate for further investigation of flowering-time regulation in cassava under various environmental conditions.

## Conclusion

The current studies provide evidence that on the basis of nodes-to-flower, cassava flowers earlier under relatively cool field and growth chamber conditions, and is delayed at warmer temperatures in the moderate temperature range of 22 to 34°C. Late flowering genotypes exhibited a plastic response to the environment as they were much more sensitive to their growth environments than early flowering genotypes and their delayed flowering time was pronounced in the warmer Ibadan field and at warmer temperatures in growth chambers. Transcriptomes under field and controlled-temperature conditions identified a set of flowering-time genes that were expressed in a temperature dependent manner. Expression of a cassava homolog of the flowering repressor gene *TEM1* in response to the tested environmental conditions was consistent with a its postulated role as a flowering inhibitor in cassava. This information advances our understanding of factors that regulate flowering in cassava, and are potentially valuable in managing genotypes and environmental conditions in breeding programs.

## Supporting information

S1 Fig Vegetative growth under field conditions. Percent plant survival in Ubiaja and Ibadan.
(TIF)

S2 Fig Vegetative growth of eight genotypes under field conditions of Ubiaja and Ibadan. a) Plant height; b) shoot fresh weight; c) root number.
(TIF)

S3 Fig Age of plants at first flowering (forking) in three genotypes under controlled temperatures. Plants which did not flower within the experimental period were omitted.
(TIF)

S4 Fig Vegetative growth of three genotypes under controlled temperatures. a) Plant height; b) shoot fresh weight; c) root number.
(TIF)

S5 Fig Age of plants at first flowering (forking) in three genotypes under controlled temperatures. Plants which did not flower within the experimental period were assigned a value of 200 DAP.
(TIF)

S6 Fig Flowering times in IITA’s diversity population of 700 cassava genotypes (Genetic Gain Population) between 2013 and 2016 in a) Ubiaja b) Ibadan. Meta-analysis of data, some of which were reported by Diebiru-Ojo (25).
(TIF)

S1 Table Differential expression of gene transcripts in cassava leaves in Ubiaja relative to Ibadan, the reference location. RNA samples were from all genotypes and sampling times in the Ubiaja and Ibadan locations, and analyzed with a full model that included these factors. Analysis was conducted using the DESeq2 package by Bioconductor. Each transcript was annotated by the best match between *Manihot esculenta* genome v6 as presented at Phytozome13.
(XLSX)

S2 Table Relative expression of differentially expressed genes in response to field location (Ibadan vs Ubiaja), genotype (‘0002 vs ‘419), and timepoint of development (pre-flowering vs post-flowering). The table shows log_2_ normalized average counts (3 biological replicates) of 1074 genes with significant (p_adj_≤0.05) differential expression among averages of biological replicates per time point, genotype and location.
(XLSX)

S3 Table Enrichment analysis for gene ontology (GO) categories that were overrepresented among the 1074 genes that were differentially expressed in leaves at the Ubiaja location relative to the Ibadan location. Arabidopsis homologs of cassava differentially expressed genes were compared to the *Arabidopsis thaliana* expression database using ShinyGO [38].
(XLSX)

S4 Table Differential expression of gene transcripts in leaves of cassava plants grown in controlled-environment cabinets. RNA samples were from all genotypes and sampling times in the Ubiaja and Ibadan locations, and analyzed with a full model that included these factors. Analysis was conducted using the DESeq2 package by Bioconductor. Each transcript was annotated by the best match between *Manihot esculenta* genome v7 as presented at Phytozome13.
(XLSX)

S5 Table Relative expression (log_2_) of differentially expressed genes in response to controlled-environment temperature (22, 28 or 34°C), genotype (‘516, ‘350, ‘2195), and timepoint of development (47 and 96 DAP). The table shows log_2_ normalized counts of 7253 genes differentially expressed (p_adj_≤0.05) (averages of biological replicates per time point, genotype and location).
(XLSX)

S6 Table Enrichment analysis for gene ontology (GO) categories that were overrepresented among the 7253 genes that were differentially expressed in leaves at 22°C relative to 28 and/or 34°C. Arabidopsis homologs of cassava differentially expressed genes were compared to the *Arabidopsis thaliana* expression database using ShinyGO [38].
(XLSX)
