# Supplementary figures and images for: Environmental responsiveness of flowering time in cassava genotypes and associated transcriptome changes
Source: PLoS One. 2021 Jul 21;16(7):e0253555. doi: 10.1371/journal.pone.0253555 (PMC8294508; doi:10.1371/journal.pone.0253555)

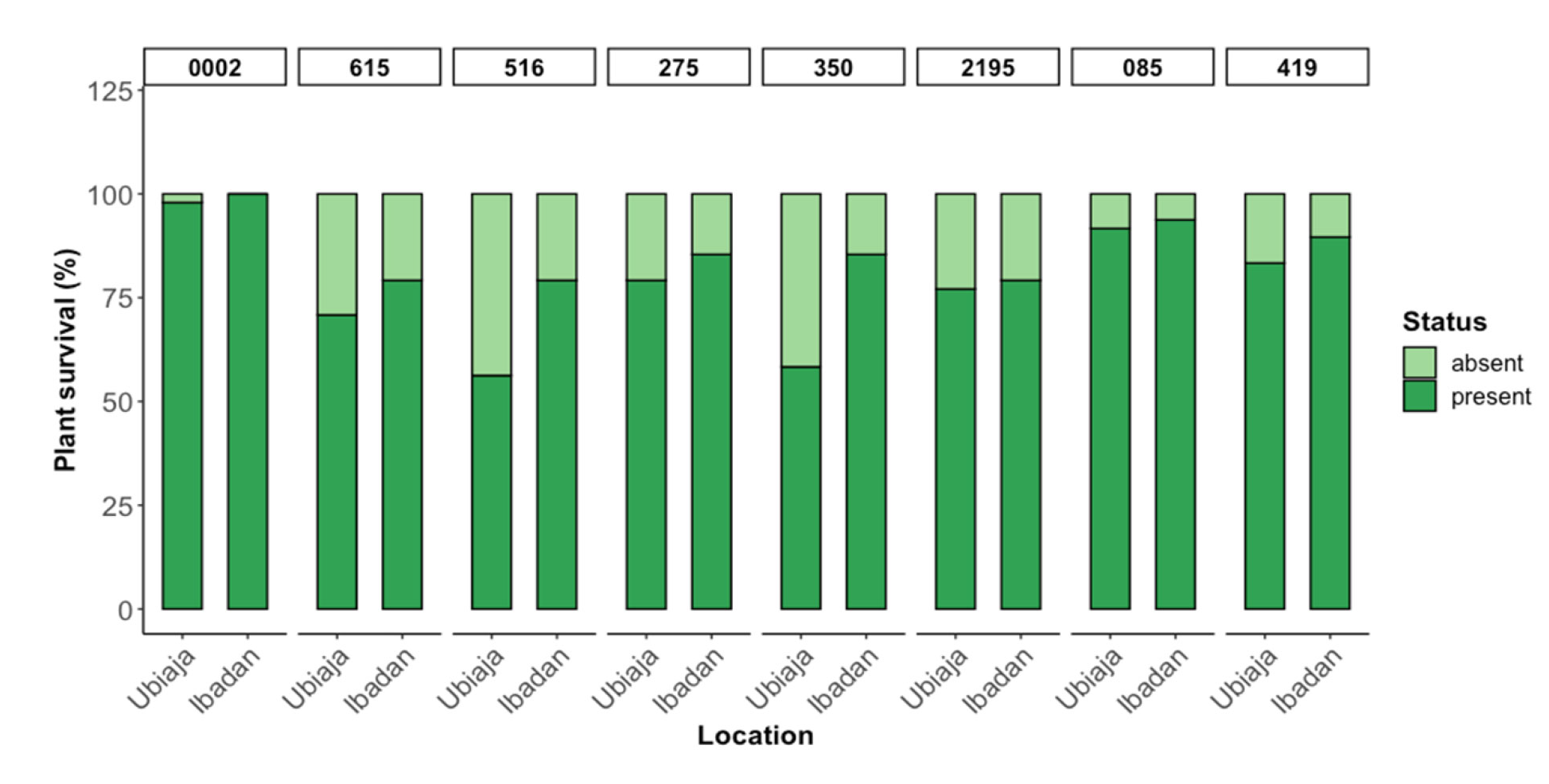

Supplement: S1 Fig — Percent plant survival in Ubiaja and Ibadan. (TIF) [file pone.0253555.s001.tif]

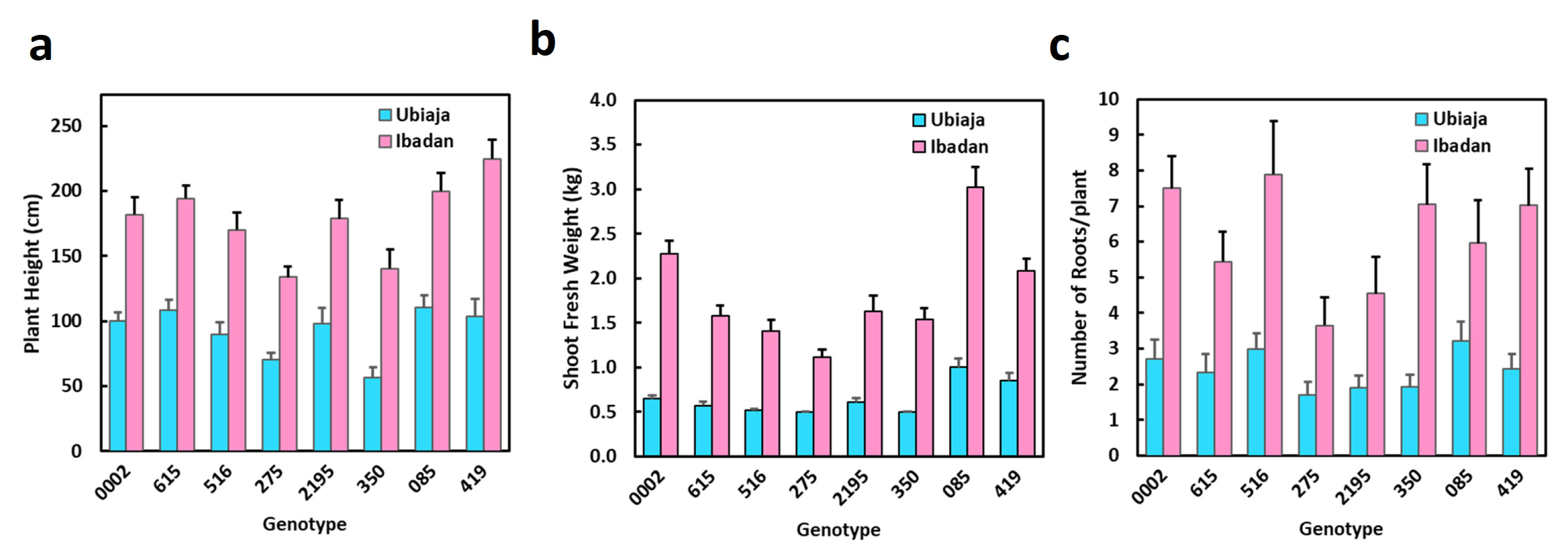

Supplement: S2 Fig — a) Plant height; b) shoot fresh weight; c) root number. (TIF) [file pone.0253555.s002.tif]

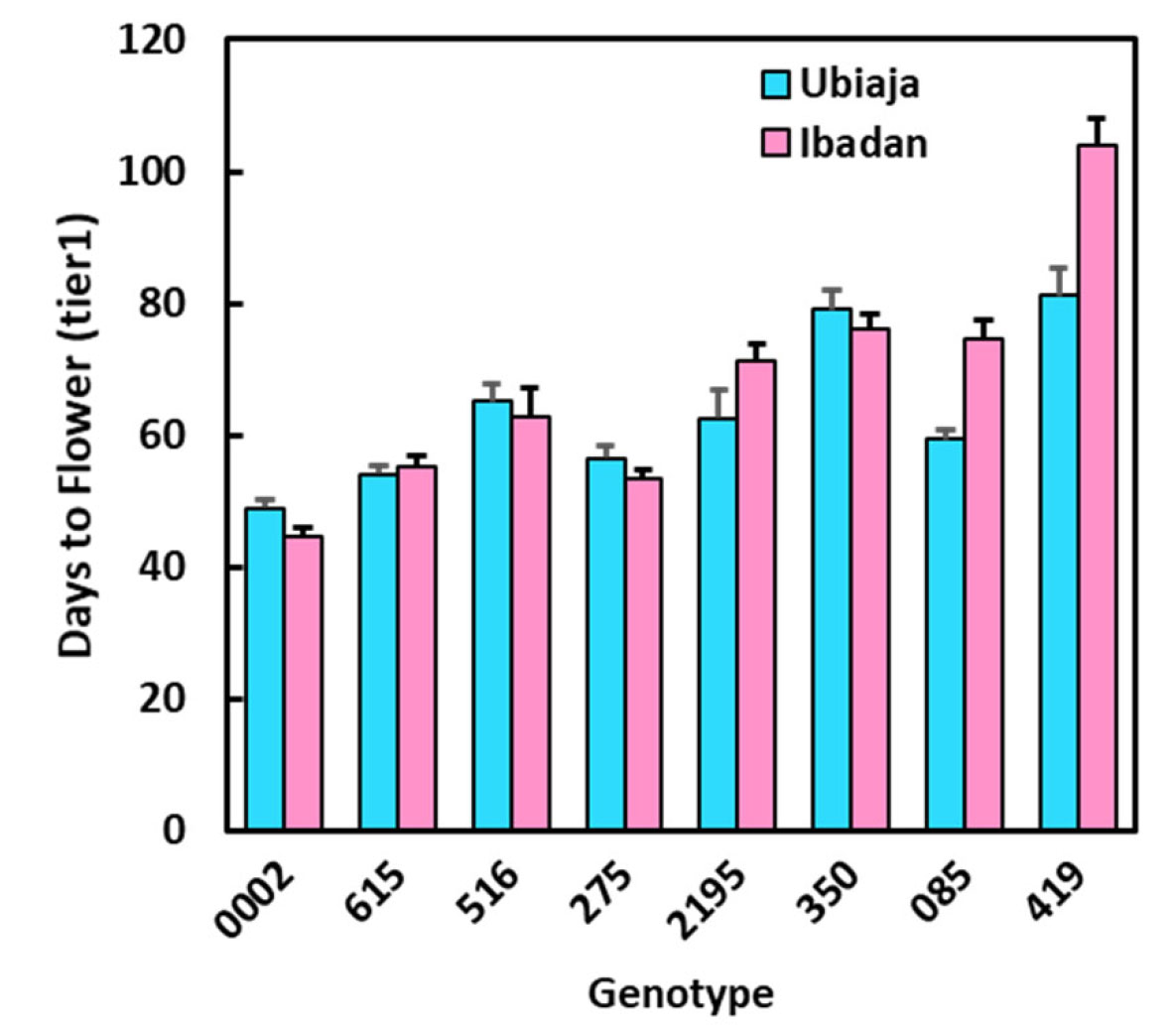

Supplement: S3 Fig — Plants which did not flower within the experimental period were omitted. (TIF) [file pone.0253555.s003.tif]

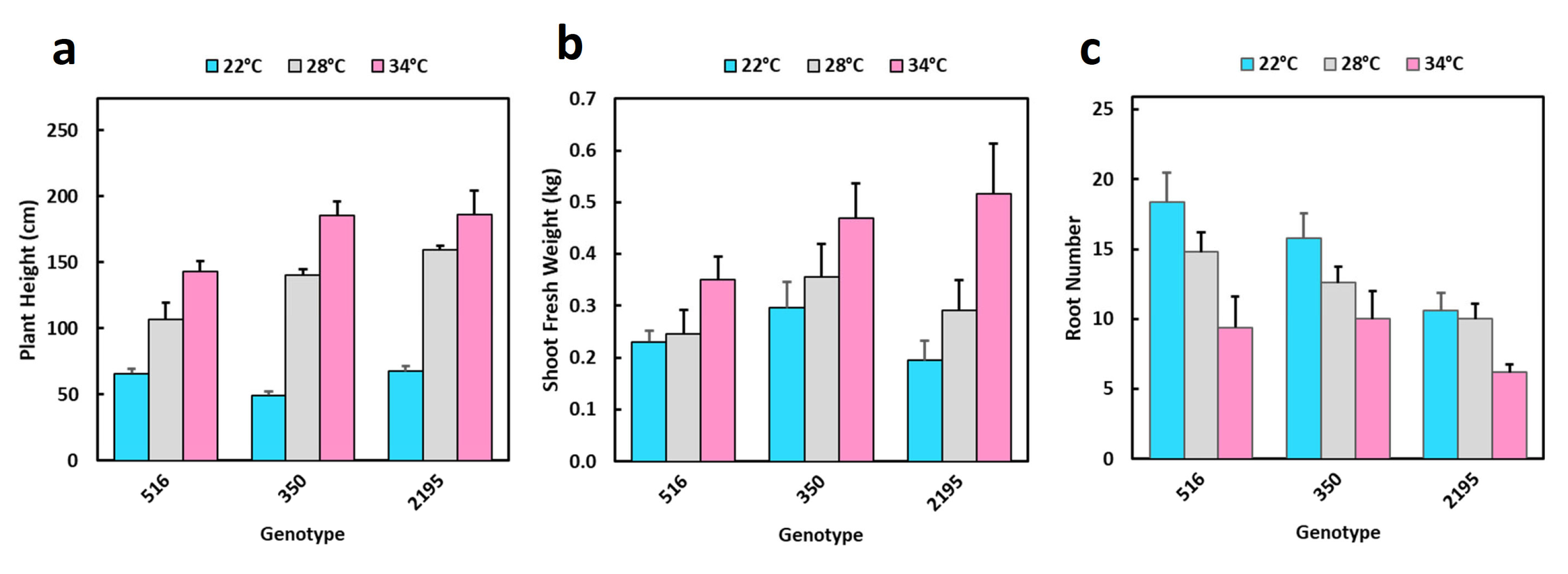

Supplement: S4 Fig — a) Plant height; b) shoot fresh weight; c) root number. (TIF) [file pone.0253555.s004.tif]

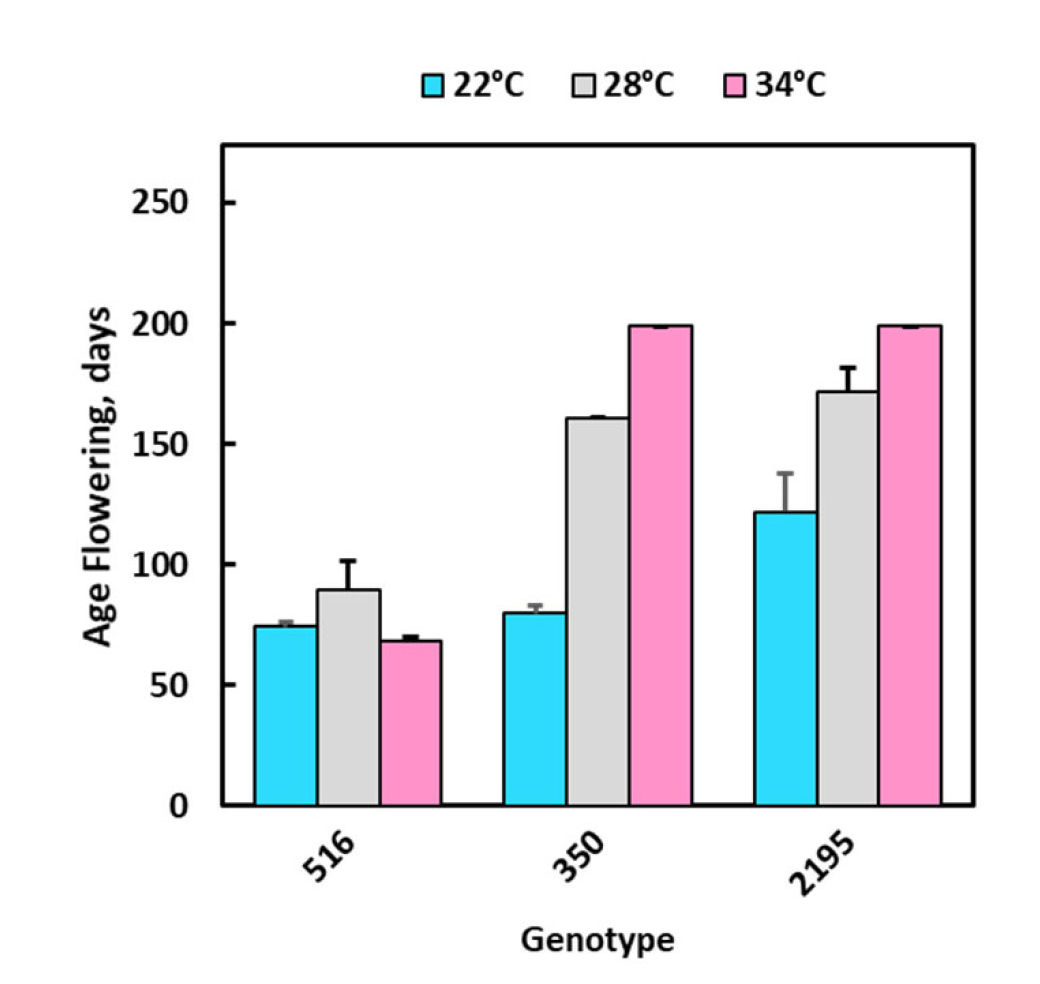

Supplement: S5 Fig — Plants which did not flower within the experimental period were assigned a value of 200 DAP. (TIF) [file pone.0253555.s005.tif]

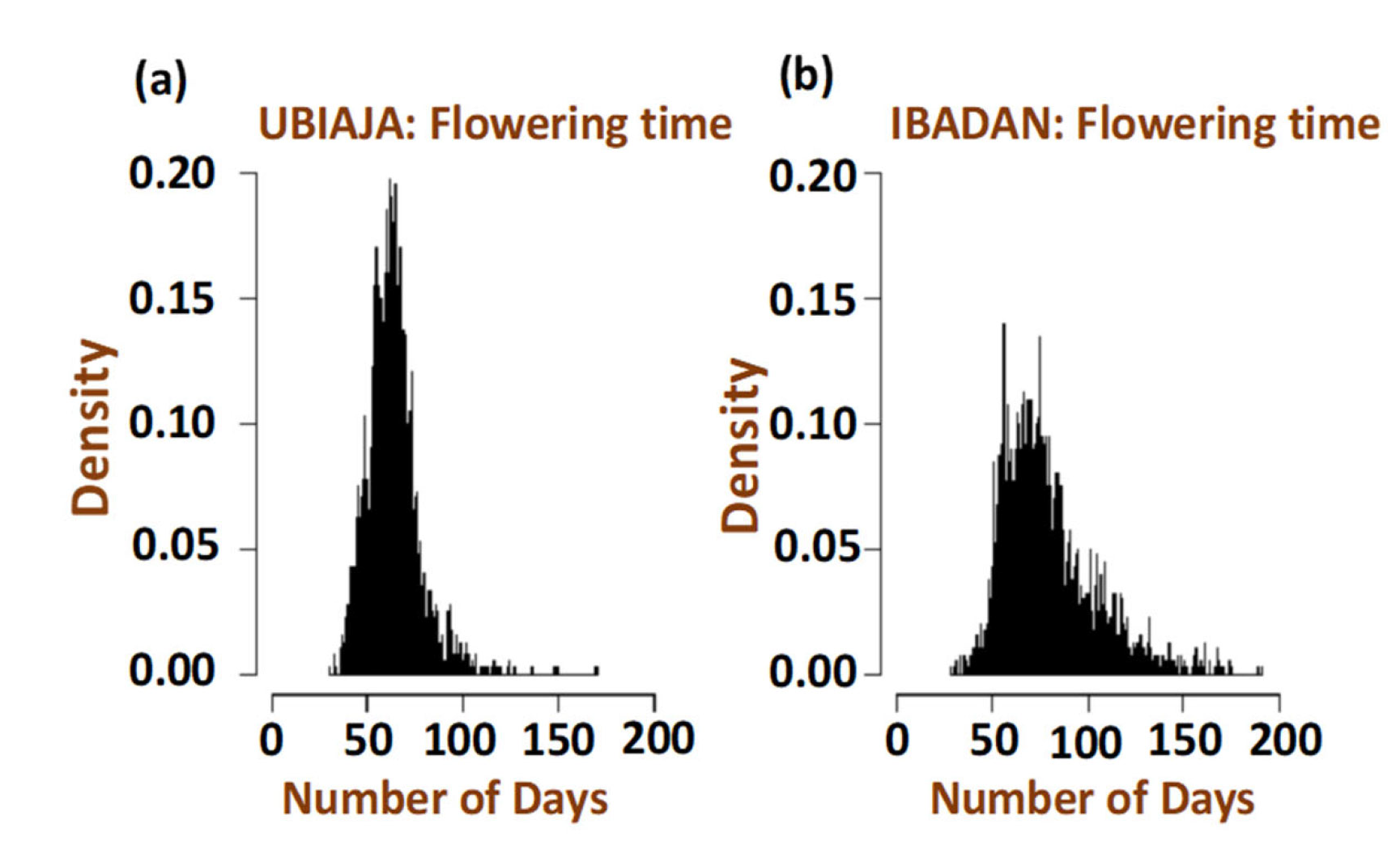

Supplement: S6 Fig — Flowering times in IITA’s diversity population of 700 cassava genotypes (Genetic Gain Population) between 2013 and 2016 in a) Ubiaja b) Ibadan. Meta-analysis of data, some of which were reported by Diebiru-Ojo (25). (TIF) [file pone.0253555.s006.tif]
